# Supplementary material for: Alcohol intake and pancreatic cancer risk: An analysis from 30 prospective studies across Asia, Australia, Europe, and North America
Source: PLoS Med. 2025 May 20;22(5):e1004590. doi: 10.1371/journal.pmed.1004590 (PMC12091891; doi:10.1371/journal.pmed.1004590)
Supplement: S1 File — Fig A: Cohort-specific associations between alcohol intake, expressed for a 10 g/day increase, and the risk of pancreatic cancer. Fig B: Pancreatic cancer hazard ratios (solid line) and corresponding 95% confidence interval (dashed line) as a function of alcohol intake (ranging from 0 to 100 g/day). Fig C: Heterogeneity in the alcohol-pancreatic cancer association, for alcohol intake expressed for a 10 g/day increase, by body mass index, diabetes status, education, follow-up time and multivitamin use. Fig D: Association between alcohol intake and the risk of pancreatic cancer by type of alcoholic beverage and by geographic region, sex, education, and smoking status. Fig E: Association between alcohol intake and the risk of pancreatic cancer using different levels of adjustment for smoking habits and pancreatic cancer risk factors. Fig F: Association between alcohol intake and the risk of pancreatic cancer among studies without (left) and with (right) information on past drinking (COSM, EPIC, HPFS, MCCS, NHS, PLCO, SMC). Table A: Study, region, and sex-specific alcoholic beverage intake among drinkers. Table B: Study institutional review board and approval number. File A: Statistical analysis plan for evaluation of the association between alcohol intake and pancreatic cancer risk within the Pooling Project on Alcohol and Cancer. (DOCX) [file pmed.1004590.s001.docx]

Manuscript - Alcohol intake and pancreatic cancer risk: an analysis from 30 prospective studies across Asia, Australia, Europe, and North America

**S1 Supporting information**

**Figures**

**Fig A.** …………………..……………………………………………………………………………………...2
Cohort-specific associations between alcohol intake, expressed for a 10 g/day increase, and the risk of pancreatic cancer

**Fig B.** …………………..……………………………………………………………………………………...3
Pancreatic cancer hazard ratios (solid line) and corresponding 95% confidence interval (dashed line) as a function of alcohol intake (ranging from 0 to 100 g/day)

**Fig C.** …………………..……………………………………………………………………………………...4
Heterogeneity in the alcohol-pancreatic cancer association, for alcohol intake expressed for a 10 g/day increase, by body mass index, diabetes status, education, follow-up time and multivitamin use.

**Fig D.** …………………..……………………………………………………………………………………...5
Association between alcohol intake and the risk of pancreatic cancer by type of alcoholic beverage and by geographic region, sex, education, and smoking status

**Fig E.** …………………..……………………………………………………………………………………...6
Association between alcohol intake and the risk of pancreatic cancer using different levels of adjustment for smoking habits and pancreatic cancer risk factors

**Fig F.** …………………..……………………………………………………………………………………...7
Association between alcohol intake and the risk of pancreatic cancer among studies without (left) and with (right) information on past drinking (COSM, EPIC, HPFS, MCCS, NHS, PLCO, SMC)

**Tables**

**Table A.** ………………...……………………………………………………………………………………….8
Study, region, and sex-specific alcoholic beverage intake among drinkers

**Table B.** ………………...………………………………………………………………………………………10
Study institutional review board and approval number

**Files**

**File A.** ………………...…………………………………………………………………………………………12

Statistical analysis plan for evaluation of the association between alcohol intake and pancreatic cancer risk within the Pooling Project on Alcohol and Cancer

**Fig A.** Cohort-specific associations between alcohol intake, expressed for a 10 g/day increase, and the risk of pancreatic cancer.

**
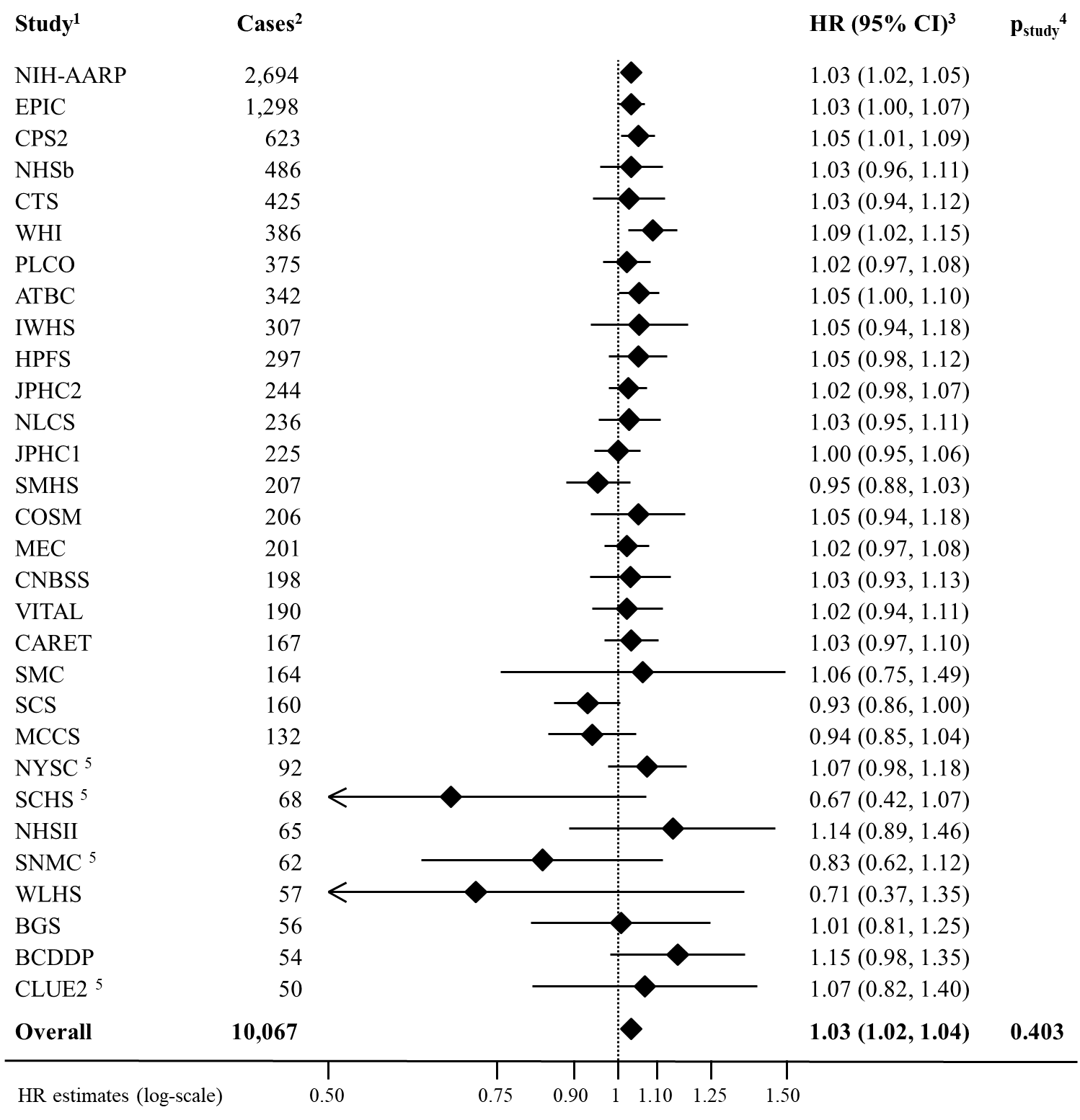
**

^1^ Abbreviations: ATBC: Alpha-Tocopherol Beta-Carotene Cancer Prevention Study; BCDDP: Breast Cancer Detection Demonstration Project Follow-Up Study; CARET: Beta-Carotene and Retinol Efficacy Trial; CLUE II: Campaign against Cancer and Heart Disease; CNBSS: Canadian National Breast Screening Study; COSM: Cohort of Swedish Men; CPS II: Cancer Prevention Study II Nutrition Cohort; CTS: California Teachers Study; EPIC: European Prospective Investigation into Cancer and Nutrition; GS: Generations Study; HPFS: Health Professionals Follow-up Study; IWHS: Iowa Women’s Health Study; JPHC I: Japan Public Health Center-based Prospective Study I; JPHC II: Japan Public Health Center-based Prospective Study II; MCCS: Melbourne Collaborative Cohort Study; MEC: Multiethnic Cohort Study; NHS: Nurses’ Health Study; NHS II: Nurses’ Health Study II; NIH-AARP: NIH-AARP Diet and Health Study; NLCS: Netherlands Cohort Study; NYSC: New York State Cohort; PLCO: Prostate, Lung, Colorectal, and Ovarian Cancer Screening Trial; SCHS: Singapore Chinese Health Study; SCS: Shanghai Cohort Study; SMC: Swedish Mammography Cohort; SMHS: Shanghai Men’s Health Study; SNMC: Swedish National March Cohort; VITAL: VITamins and Lifestyle Study: Cohort Study of Dietary Supplements and Cancer Risk; WHI: Women’s Health Initiative; WLHS: Women’s Lifestyle and Health Study. ^2^ Cohorts were sorted by number of cases; ^3^ Cox proportional hazard models were adjusted for smoking status, smoking duration, smoking intensity, time since smoking cessation, diabetes status, BMI, height, education, race and ethnicity, and physical activity and an indicator variable for alcohol drinking status (0: <0.1 g/day, 1: ≥ 0.1 g/day). The model was stratified by age at baseline (in 1-year categories), year of baseline questionnaire completion (in 1-calendar-year categories), cohort, country (in EPIC) and sex. Interaction terms between a 10 g/day increase in alcohol intake and each study were included in the model; ^4^ Heterogeneity across studies was tested comparing the Wald test statistics for significance to a χ^2^ distribution with 29 degrees of freedom; ^5^ Women in NYSC were not included because the number of pancreatic cancer cases was lower than 50. Women in SCHS were not included because the prevalence of drinking at baseline was lower than 10%; Men in SNMC and CLUE II were not included because the number of pancreatic cancer cases was lower than 50;

**Fig B.** Pancreatic cancer hazard ratios (solid line) and corresponding 95% confidence interval (dashed line) as a function of alcohol intake (ranging from 0 to 100 g/day) ^1^_._


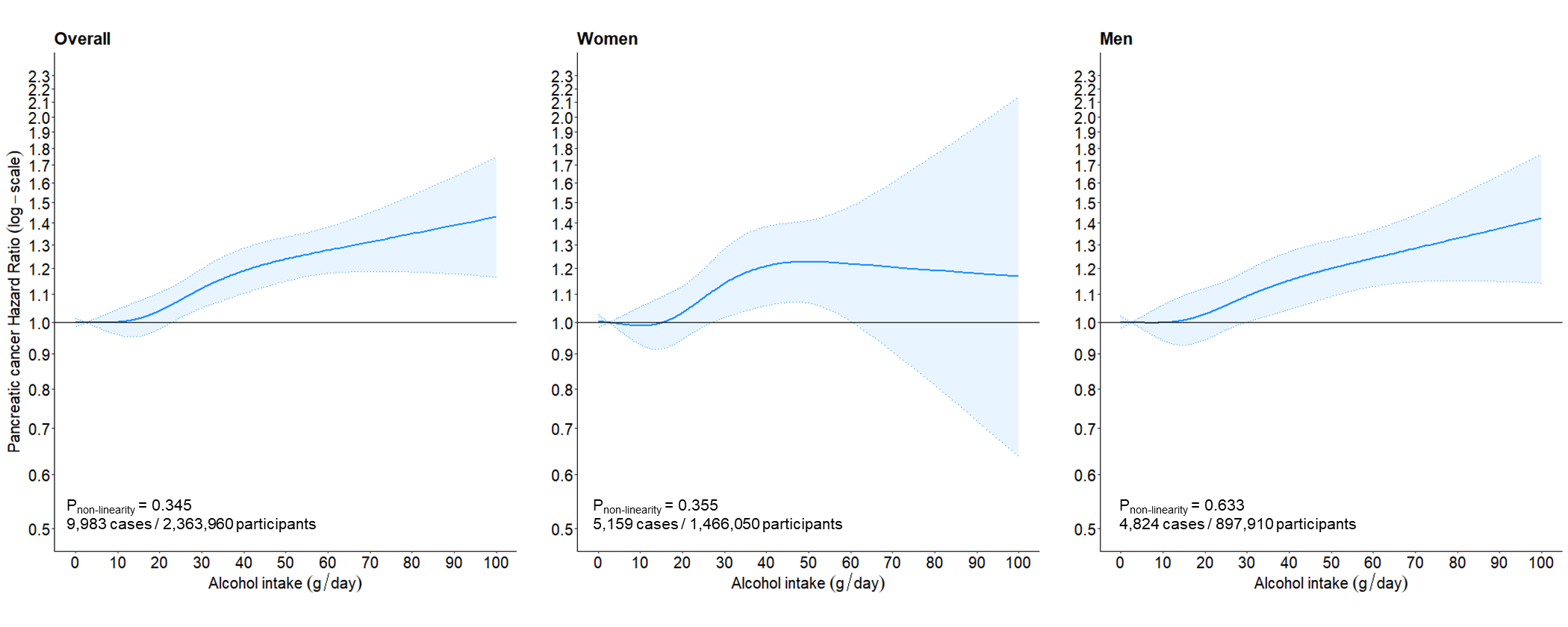


^1^ Hazard ratios estimated in Cox models including restricted cubic splines with four internal knots placed at alcohol intake of 5, 15, 30 and 60 g/day and using 2.5 g/day as the reference, after excluding participants with alcohol intake >100 g/day. Departure from linearity was evaluated by comparing the difference in log-likelihood of models with and without non-linear terms to a χ² distribution with two degrees of freedom. Models were adjusted for smoking status, smoking duration, smoking intensity, time since smoking cessation, diabetes status, BMI, height, education, race and ethnicity, physical activity and an indicator variable for alcohol drinking status and stratified by age at baseline (in 1-year categories), year of baseline questionnaire completion (in 1-calendar-year categories), cohort, country (in EPIC) and sex.

**Fig C.** Heterogeneity in the alcohol-pancreatic cancer association, for alcohol intake expressed for a 10 g/day increase, by body mass index, diabetes status, education, follow-up time and multivitamin use.


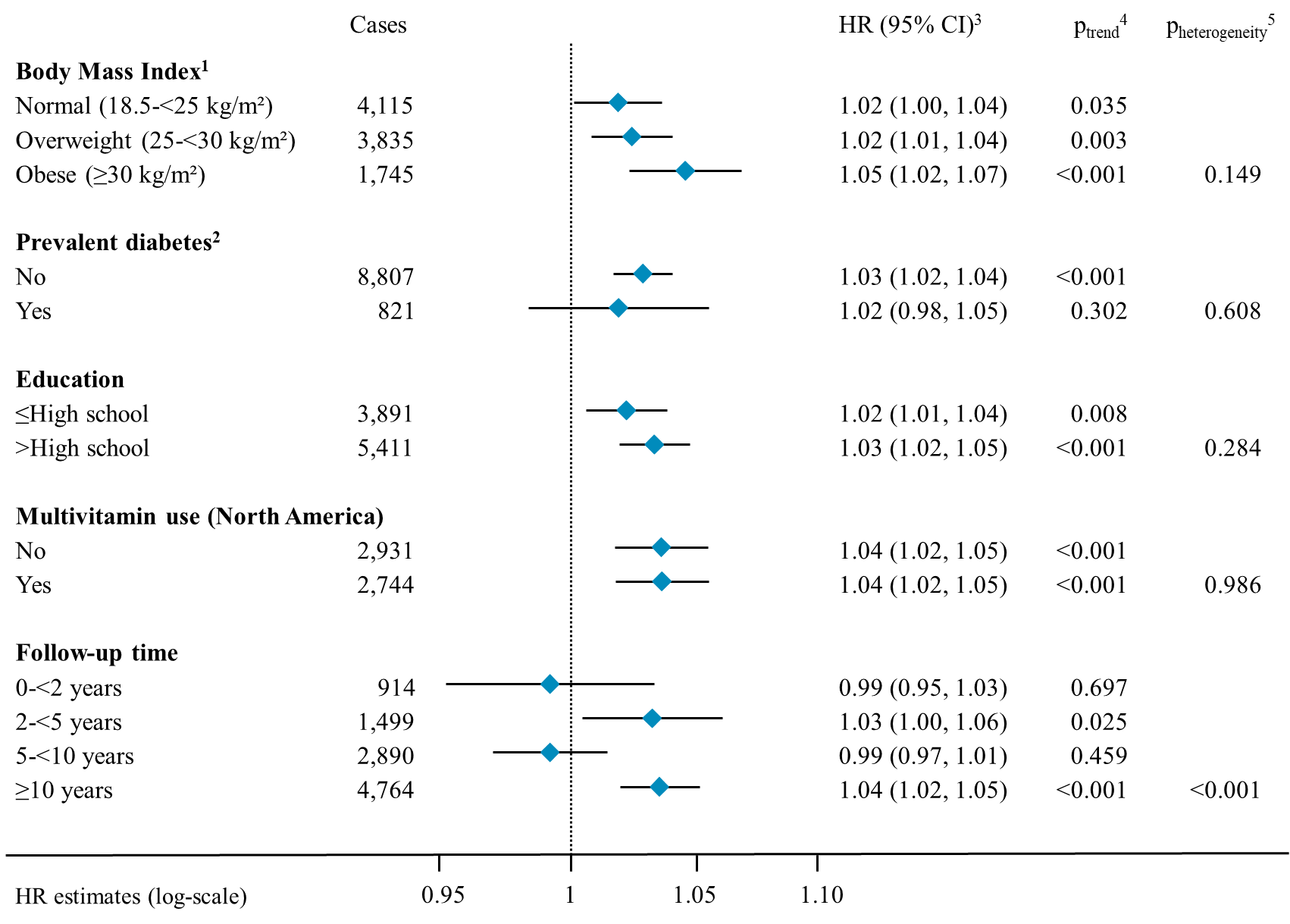


^1^ Participants with BMI <18.5 kg/m² were excluded; ^2^ Self-reported diabetes status included patients with either type-I or type-II diabetes; ^3^ Cox proportional hazard models were adjusted for smoking status, smoking duration, smoking intensity, time since smoking cessation, diabetes status, BMI, height, education, race and ethnicity, physical activity, and an indicator variable for alcohol drinking status. Models were stratified by age at baseline, year of baseline questionnaire completion, study, country (in EPIC) and sex. BMI, diabetes or education were not included as covariates when they were the factor under evaluation for the heterogeneity test. Models included interaction terms between alcohol intake modeled in continuous and the factor under evaluation, while participants without information for the factor under evaluation were excluded; ^4^ P-value for alcohol consumption modelled in continuous in a model including an indicator variable expressing alcohol drinking status; ^5^ Heterogeneity by either BMI, diabetes, education, multivitamin use or follow-up time were obtained by comparing the log-likelihood of models with and without the interaction terms to a χ^2^ distribution with degrees of freedom equal to the number of modifier categories minus one.

**Fig D.** Association between alcohol intake and the risk of pancreatic cancer by type of alcoholic beverage and by geographic region, sex, education, and smoking status.

**
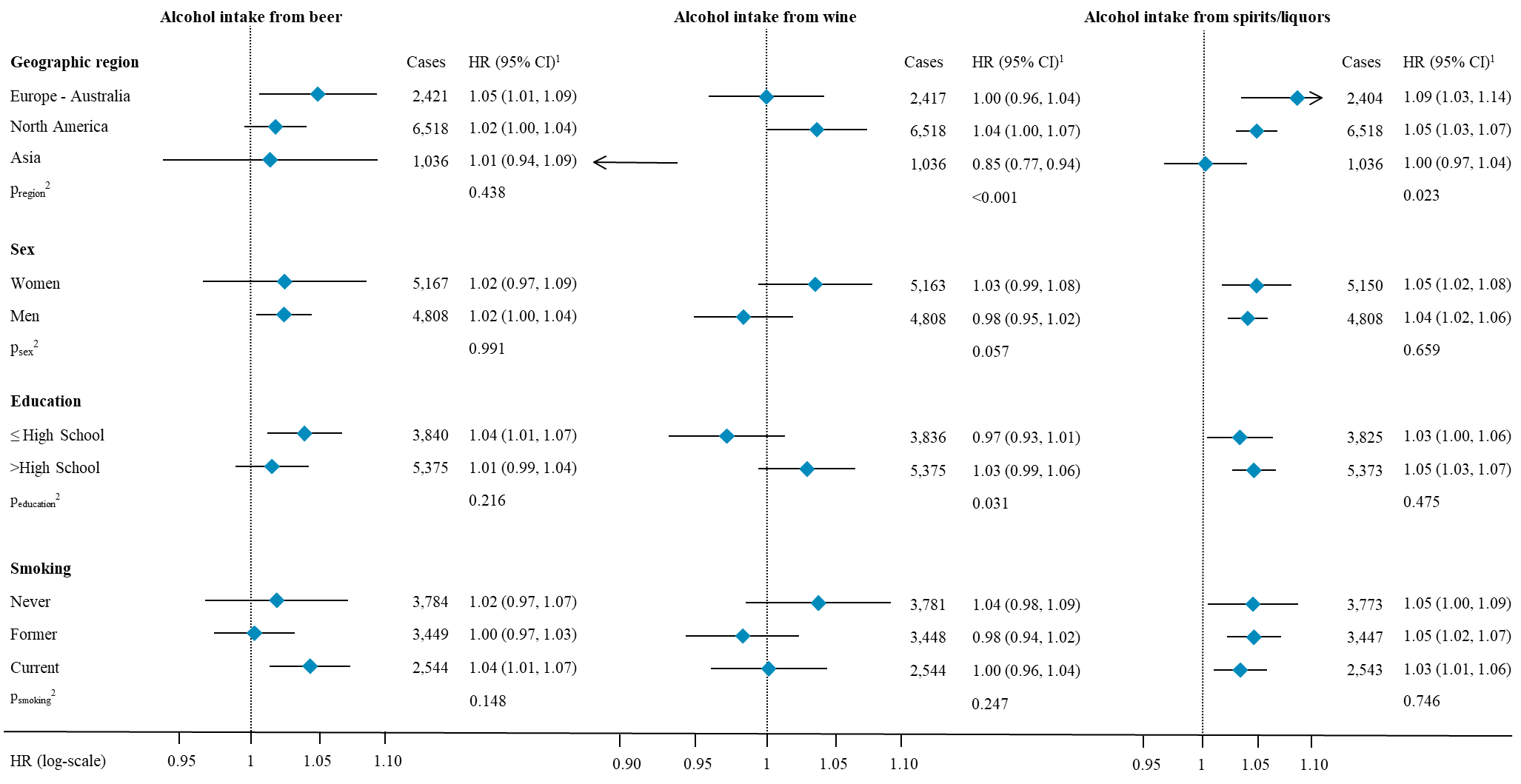
**

^1^ Hazard ratio (HR) for a 10 g/day increase in alcohol intake from Cox proportional hazard models were adjusted for alcohol intake from the alcoholic beverages other than the one under evaluation, smoking status, smoking duration, smoking intensity, time since smoking cessation, diabetes status, BMI, height, education, race and ethnicity, physical activity and an indicator variable for alcohol drinking status based on total alcohol intake. Smoking status or education were not included as covariates when they were the factor under evaluation for the heterogeneity test. Models were stratified by age at baseline, year of baseline questionnaire completion, cohort, country (in EPIC) and sex;^2^ Heterogeneity by geographic region/sex/education/smoking level for a given type of beverage was tested adding interaction terms between the type of beverage and the factor under evaluation, then comparing the Wald test statistics for significance to a χ^2^ distribution with one (sex and education) or two (geographic region, smoking status) degrees of freedom, in a model including an indicator variable expressing alcohol drinking status.

**Fig E.** Association between alcohol intake and the risk of pancreatic cancer using different levels of adjustment for smoking habits and pancreatic cancer risk factors


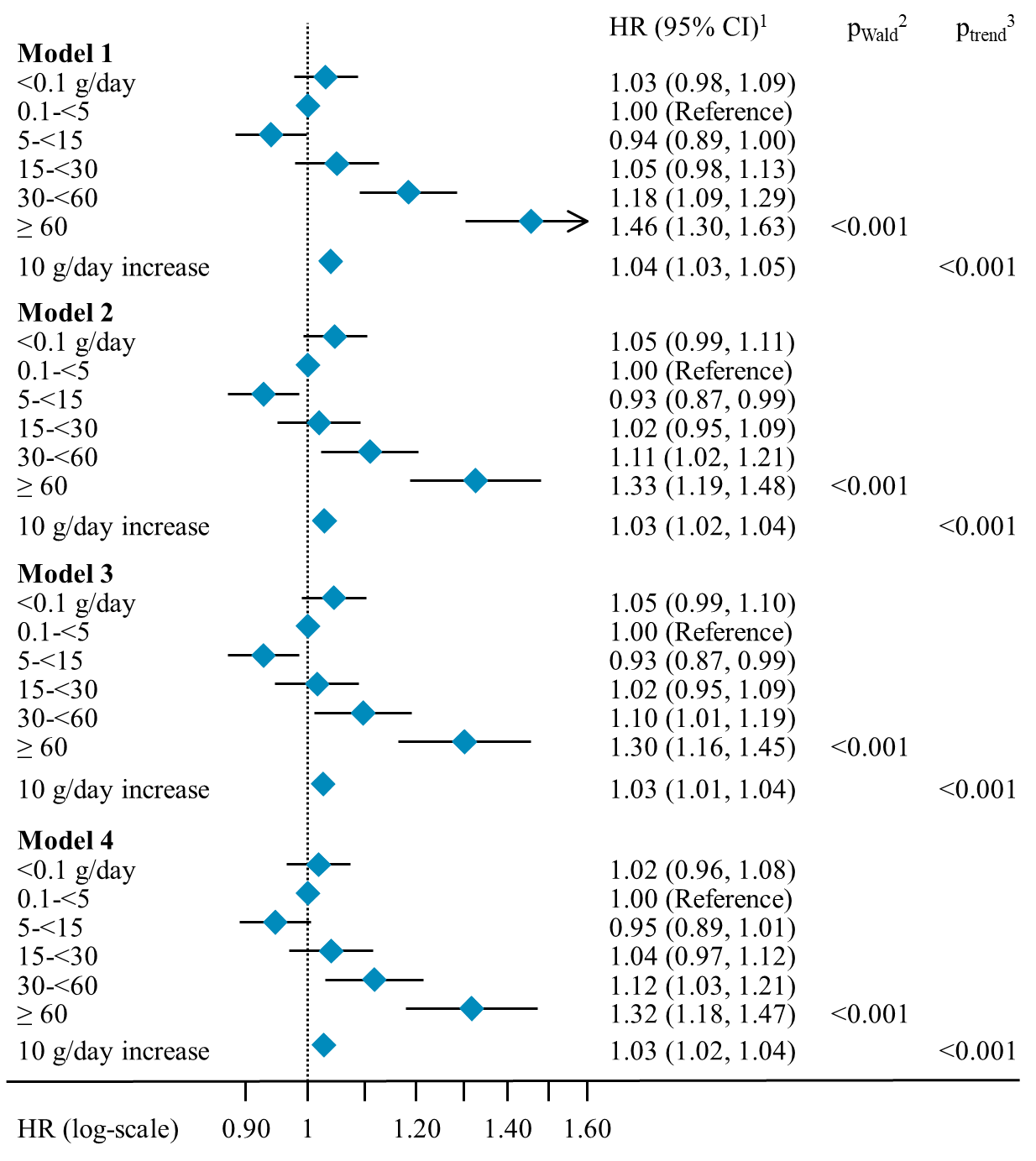


^1^ All models were stratified by age at baseline, year of baseline questionnaire completion, study, country (in EPIC), and sex. Analyses in continuous were all adjusted for an indicator variable for alcohol drinking status. **Model 1** included no covariates. **Model 2** was adjusted for smoking status. **Model 3** was Model 2 further adjusted for smoking duration, smoking intensity, and time since smoking cessation. **Model 4** was Model 3 further adjusted for diabetes status, BMI, height, education, race and ethnicity, and physical activity; ^2^ P-value for the Wald test statistics compared with a χ^2^ distribution with degrees of freedom equal to the number of alcohol intake categories minus one, not including the category of non-drinkers (<0.1 g/day); ^3^ P-value for alcohol consumption modelled as a continuous variable for a 10 g/day increase, with inclusion in the model of an indicator variable expressing the alcohol drinking status.

**Fig F.** Association between alcohol intake and the risk of pancreatic cancer among studies without (left) and with (right) information on past drinking (COSM, EPIC, HPFS, MCCS, NHS, PLCO, SMC)


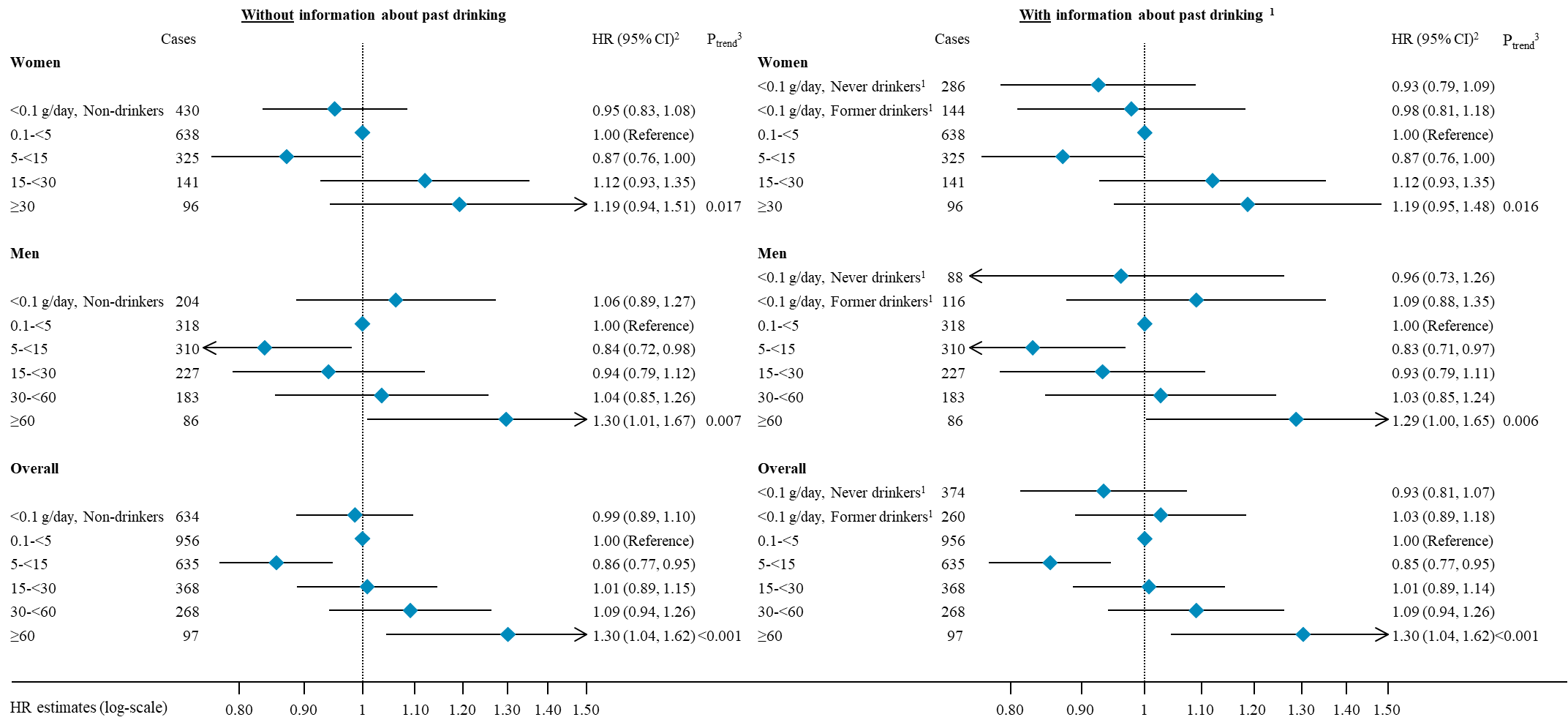


^1^ Non-drinkers category (<0.1 g/day) was divided into never drinkers and former drinkers; ^2^ Cox proportional hazard models were adjusted for smoking status, smoking duration, smoking intensity, time since smoking cessation, diabetes status, BMI, height, education, race and ethnicity, and physical activity. Models were stratified by age at baseline, year of baseline questionnaire completion, study, country (in EPIC), and sex; ^3^ P-value for the Wald test statistics compared with a χ^2^ distribution with 4 degrees of freedom (except for women, 3 degrees of freedom), using the category of light drinkers (0.1-<5 g/day) as reference.

**Table A.** Study, region, and sex-specific alcoholic beverage intake among drinkers

| Study^†^ | Country / Continent | Sex^†^ | Alcohol drinkers (%)^‡^ | Total alcohol intake among drinkers (g/day) ^‡^ | Beer drinkers (%)^‡^ | Alcohol intake from beer among drinkers (g/day) ^‡^ | Wine drinkers (%)^‡^ | Alcohol intake from wine among drinkers (g/day) ^‡^ | Spirit/liquor drinkers (% )^‡^ | Alcohol intake from spirits/liquors among drinkers (g/day) ^‡^ |
| --- | --- | --- | --- | --- | --- | --- | --- | --- | --- | --- |
| Europe/Australia |  |  |  |  |  |  |  |  |  |  |
| ATBC | Finland | M | 89 | 13.0 (1.6, 45.7) | 71 | 4.6 (0.8, 23.1) | 21 | 2.2 (0.4, 9.6) | 81 | 8.0 (0.6, 23.5) |
| COSM | Sweden | M | 92 | 9.0 (1.6, 23.6) | 87 | 5.0 (0.9, 15.4) | 68 | 2.4 (0.5, 7.3) | 74 | 1.8 (0.4, 4.9) |
| EPIC | Europe (a) | M+W | 85 | 6.9 (0.7, 34.3) | 55 | 1.8 (0.2, 14.2) | 78 | 4.8 (0.3, 24.4) | 43 | 0.9 (0.2, 6.6) |
| GS | England | W | 79 | 14.0 (5.0, 36.0) | 22 | 6.0 (3.0, 19.0) | 72 | 12.0 (2.0, 31.0) | 28 | 3.0 (2.0, 13.0) |
| MCCS | Australia | M+W | 61 | 12.7 (1.1, 43.9) | 34 | 3.0 (0.2, 26.1) | 52 | 8.6 (1.0, 30.0) | 29 | 1.4 (0.3, 9.6) |
| NLCS^§^ | Netherlands | M+W | 77 | 8.7 (0.9, 32.1) | 33 | 2.3 (0.3, 14.8) | 59 | 3.7 (0.6, 20.5) | 37 | 8.4 (0.9, 25.1) |
| SMC | Sweden | W | 82 | 3.2 (0.4, 10.2) | 60 | 1.4 (0.2, 5.0) | 73 | 2.0 (0.3, 5.7) | 46 | 0.2 (0.2, 2.5) |
| SNMC | Sweden | M+W | 87 | 6.3 (0.8, 23.1) | 64 | 2.7 (0.2, 7.9) | 80 | 3.9 (0.6, 18.6) | 53 | 0.2 (0.2, 2.1) |
| WLHS | Sweden | W | 86 | 2.9 (0.5, 9.0) | 70 | 1.3 (0.2, 4.8) | 68 | 1.3 (0.3, 5.3) | 39 | 0.4 (0.2, 3.0) |
|  | **All women** |  | 82 | 5.7 (0.6, 25.0) | 44 | 1.4 (0.2, 7.2) | 75 | 4.8 (0.4, 22.2) | 34 | 0.9 (0.2, 5.8) |
|  | **All men** |  | 92 | 12.1 (1.6, 45.8) | 80 | 4.8 (0.6, 21.7) | 71 | 4.5 (0.4, 29.8) | 71 | 1.7 (0.2, 11.2) |
|  | **All Europe/Australia** |  | 85 | 7.1 (0.8, 32.0) | 54 | 2.3 (0.3, 13.5) | 74 | 4.8 (0.4, 23.7) | 44 | 1.1 (0.2, 8.3) |
| North America |  |  |  |  |  |  |  |  |  |  |
| BCDDP | USA | W | 49 | 3.5 (0.4, 20.1) | 17 | 0.9 (0.2, 7.4) | 38 | 1.3 (0.2, 11.6) | 30 | 2.2 (0.3, 15.5) |
| CARET | USA | W | 65 | 10.6 (1.0, 55.2) | 40 | 5.1 (0.8, 45.3) | 38 | 2.3 (1.0, 18.7) | 41 | 5.5 (0.8, 32.5) |
| CLUE II | USA | W | 33 | 2.6 (0.9, 16.5) | 16 | 1.9 (0.5, 13.6) | 21 | 1.0 (0.5, 6.0) | 15 | 1.7 (0.7, 15.5) |
| CNBSS | Canada | W | 77 | 6.6 (1.0, 27.7) | 26 | 1.7 (0.4, 9.3) | 67 | 3.1 (0.5, 15.7) | 51 | 2.6 (0.5, 15.9) |
| CPS II | USA | M+W | 58 | 6.7 (0.9, 35.7) | 31 | 1.9 (0.9, 13.0) | 41 | 1.9 (0.7, 13.5) | 34 | 6.0 (0.9, 35.1) |
| CTS | USA | W | 64 | 7.5 (3.2, 22.2) | 26 | 3.8 (3.8, 13.2) | 58 | 3.2 (2.6, 11.1) | 31 | 4.3 (4.3, 15) |
| HPFS | USA | M | 76 | 9.6 (1.8, 36.9) | 55 | 1.8 (1.0, 12.8) | 59 | 1.8 (0.9, 10.3) | 52 | 6.0 (1.1, 35.0) |
| IWHS | USA | W | 45 | 3.4 (0.9, 21.3) | 18 | 1.8 (1.1, 13.2) | 30 | 1.7 (0.9, 6.2) | 26 | 2.1 (1.2, 15.1) |
| MEC | USA | M+W | 64 | 9.7 (0.9, 47.6) | 38 | 3.7 (0.4, 32.4) | 48 | 3.2 (0.4, 22.6) | 32 | 4.3 (0.5, 32.9) |
| NHS | USA | W | 64 | 4.7 (0.9, 27.5) | 21 | 1.8 (1.0, 12.8) | 53 | 1.8 (0.9, 11.0) | 37 | 2.0 (1.1, 14.0) |
| NHS II | USA | W | 57 | 2.8 (0.9, 12.5) | 34 | 2.0 (0.9, 9.0) | 43 | 1.5 (0.9, 6.2) | 21 | 1.2 (1.2, 6.5) |
| NIH-AARP | USA | M+W | 75 | 4.5 (0.5, 37.3) | 51 | 1.3 (0.2, 15.6) | 60 | 1.1 (0.2, 14.4) | 53 | 1.5 (0.3, 21.5) |
| NYSC | USA | M | 89 | 4.8 (0.2, 34.2) | 0 | , ^§^ | 0 | , ^§^ | 0 | , ^§^ |
| PLCO | USA | M+W | 72 | 4.1 (0.6, 28.6) | 48 | 1.4 (0.2, 14.8) | 59 | 0.8 (0.3, 10.8) | 50 | 0.9 (0.3, 19.3) |
| VITAL | USA | M+W | 64 | 6.8 (0.8, 32.5) | 38 | 1.8 (0.8, 14.6) | 52 | 3.1 (0.7, 16.5) | 32 | 2.0 (0.8, 20.9) |
| WHI | USA | W | 58 | 4.6 (0.8, 23.6) | 20 | 0.8 (0.4, 5.0) | 51 | 2.3 (0.5, 16.5) | 26 | 2.0 (0.4, 13.9) |
|  | **All women** |  | 62 | 4.0 (0.7, 22.2) | 25 | 1.2 (0.2, 7.4) | 52 | 1.9 (0.5, 12.4) | 35 | 1.5 (0.3, 15.0) |
|  | **All men** |  | 76 | 8.3 (0.9, 45.2) | 57 | 1.9 (0.4, 19.3) | 53 | 1.5 (0.5, 15.2) | 49 | 1.9 (0.4, 26.1) |
|  | **All North America** |  | 67 | 5.6 (0.7, 32.5) | 38 | 1.7 (0.4, 13.2) | 52 | 1.8 (0.5, 13.5) | 40 | 1.6 (0.4, 20.9) |
| Asia |  |  |  |  |  |  |  |  |  |  |
| JPHC I | Japan | M+W | 45 | 23.0 (1.6, 72.0) | 25 | 11.5 (0.8, 23.0) | 2 | 1.3 (0.2, 6.0) | 16 | 28.3 (2.6, 72.0) |
| JPHC II | Japan | M+W | 43 | 23.0 (1.6, 74.6) | 27 | 11.5 (0.8, 23.0) | 4 | 1.5 (0.2, 9.0) | 17 | 28.3 (2.6, 72.0) |
| SCHS | Singapore | M | 31 | 4.4 (0.4, 28.1) | 28 | 3.8 (0.4, 27.0) | 2 | 1.6 (0.4, 11.0) | 9 | 1.5 (0.4, 10.8) |
| SCS | China | M | 42 | 21.5 (4.2, 64.5) | 19 | 5.0 (1.2, 17.5) | 24 | 13.0 (2.6, 30.2) | 20 | 21.5 (6.1, 64.5) |
| SMHS | China | M | 32 | 26.0 (8.9, 69.8) | 17 | 7.7 (3.3, 27.1) | 23 | 15.6 (4.5, 52.1) | 9 | 35.7 (7.1, 93.8) |
|  | **All women** |  | 18 | 4.9 (0.8, 23.6) | 12 | 2.5 (0.8, 18.1) | 3 | 0.9 (0.2, 6.0) | 4 | 7.7 (0.7, 38.0) |
|  | **All men** |  | 45 | 24.6 (2.9, 72.0) | 26 | 9.6 (1.5, 27.0) | 13 | 13.0 (3.0, 43.2) | 17 | 25.0 (2.6, 72.0) |
|  | **All Asia** |  | 38 | 23.0 (2.0, 71.4) | 23 | 9.0 (1.1, 26.8) | 11 | 12.1 (1.7, 39.1) | 13 | 23.8 (1.8, 72.0) |
| All regions | **Total women** |  | 68 | 5.0 (0.7, 24.0) | 32 | 1.4 (0.2, 7.5) | 59 | 2.7 (0.4, 16.5) | 34 | 1.2 (0.2, 11.4) |
|  | **Total men** |  | 75 | 10.7 (1.0, 49.1) | 58 | 3.5 (0.6, 23.0) | 51 | 2.5 (0.5, 20.4) | 49 | 1.9 (0.4, 25.8) |
|  | **Total participants** |  | 70 | 6.6 (0.8, 34.3) | 42 | 1.9 (0.3, 14.6) | 56 | 2.6 (0.4, 17.0) | 39 | 1.6 (0.3, 18.4) |

^†^ Abbreviations: ATBC: Alpha-Tocopherol Beta-Carotene Cancer Prevention Study; BCDDP: Breast Cancer Detection Demonstration Project Follow-Up Study; BGS: Breakthrough Generations Study; CARET: Beta-Carotene and Retinol Efficacy Trial; CLUE2: Campaign against Cancer and Heart Disease; CNBSS: Canadian National Breast Screening Study; COSM: Cohort of Swedish Men; CPS2: Cancer Prevention Study II Nutrition Cohort; CTS: California Teachers Study; EPIC: European Prospective Investigation into Cancer and Nutrition; HPFS: Health Professionals Follow-up Study; IWHS: Iowa Women’s Health Study; JPHC1: Japan Public Health Center-based Prospective Study I; JPHC2: Japan Public Health Center-based Prospective Study II; MCCS: Melbourne Collaborative Cohort Study; MEC: Multiethnic Cohort Study; M: Men; NHS: Nurses’ Health Study; NHSII: Nurses’ Health Study II; NIH-AARP: NIH-AARP Diet and Health Study; NLCS: Netherlands Cohort Study; NYSC: New York State Cohort; PLCO: Prostate, Lung, Colorectal, and Ovarian Cancer Screening Trial; SCHS: Singapore Chinese Health Study; SCS: Shanghai Cohort Study; SMC: Swedish Mammography Cohort; SMHS: Shanghai Men’s Health Study; SNMC: Swedish National March Cohort; VITAL: VITamins and Lifestyle Study: Cohort Study of Dietary Supplements and Cancer Risk; WHI: Women’s Health Initiative; WLHS: Women’s Lifestyle and Health Study; W: women; ^‡^ Median (10^th^-90^th^ percentiles) for continuous variables and percentage for proportion of drinkers of the beverage under evaluation; ^§^ Information on the type of alcoholic beverage was not available in NYSC; ^§^NLCS was analysed as a case-cohort study with a sub-cohort size of 3,849 participants who had active follow-up. Data are only available for the subcohort and pancreatic cancer cases. Therefore, the percentages are representative of the subcohort.

**Table B**. Study institutional review board and approval reference number

| Study | Institutional Review Board (IRB) | Approval reference number |
| --- | --- | --- |
| ATBC | Institutional Review Board of the National Cancer Institute and the Finnish National Public Health Institute | OH95CN012 |
| BCDDP | Institutional Review Board of the National Cancer Institute | 19-NCI-00784 |
| GS | South East Multi-centre Research Ethics Committee | MREC 03/01/014 |
| CARET | Institutional Review Board of the Fred Hutchinson Cancer Center | FHIRB0008673 |
| CLUE II | The Johns Hopkins Bloomberg School of Public Health and the Tufts University Health Sciences Campus Institutional Review Boards | IRB00005798 and IRB00018528 |
| CNBSS | The University of Toronto’s Human Experimentation Committee and the Women’s College Hospital research ethics board | 2007-0025-B |
| COSM | The Regional Ethical Review Board in Stockholm | 2005/861-32 |
| CPS II | The Emory University Institutional Review Board | AM14_IRB00045780 |
| CTS | The Institutional Review Board at City of Hope | No. 7224 |
| EPIC | The International Agency for Research on Cancer Ethics Committee and all local ethics committees | IARC-IEC-10052017-17-17 and IARC-PF1-032017-170215 |
| HPFS | The Institutional review boards of the Brigham and Women’s Hospital and the Harvard T.H. Chan School of Public Health | IRB24-0407: 10162: Core C: Cohort Follow-up & Database Management |
| IWHS | The University of Minnesota Institutional Review Boards | 8407M01734 and CR00016552 |
| JPHC I | The Institutional Review Board of the National Cancer Center, Tokyo, Japan | No. 2011-044 |
| JPHC II | The Institutional Review Board of the National Cancer Center, Tokyo, Japan | No. 2015-312 |
| MCCS | The Cancer Council Victoria Human Research Ethics Committee | IEC 9001 |
| MEC | The University of Southern California’s and the University of Hawaii’s Institutional Review Boards | HS-17-00714 and 2023-00178 |
| NHS | The institutional review boards of the Brigham and Women’s Hospital and the Harvard T.H. Chan School of Public Health, and those of participating registries as required. | 1999P011114 |
| NHS II | The institutional review boards of the Brigham and Women’s Hospital and the Harvard T.H. Chan School of Public Health, and those of participating registries as required. | 1999P003389 |
| NIH-AARP | The Special Studies Institutional Review Board of the U.S. National Cancer Institute | #000195 |
| NLCS | The Maastricht University Medical Center Medical Ethical Committee | MEC 85-012.1 |
| NYSC | The Institutional Review Board of the University at Buffalo, State University of New York | 406052-3 |
| PLCO | Each of the 10 screening centers and the United States National Institutes of Health Institutional review boards | OH97CN041 |
| SCHS | The National University of Singapore Institutional Review Board | STUDY19080227 and L04-026 |
| SCS | The Institutional Review Boards at the University of Minnesota and the Shanghai Cancer Institute | STUDY19080227 |
| SMC | The Regional Ethical Review Board in Stockholm | 2005/861-32 |
| SMHS | The study protocols were approved by the Institutional Review Boards of Vanderbilt University and the Shanghai Cancer Institute | IRB#000598 |
| SNMC | The ethical review board in Stockholm | 1997-205 and 2017/796-31 |
| VITAL | The institutional review board of the Fred Hutchinson Cancer Research Center | No.4343 |
| WHI | The Fred Hutchinson Cancer Center institutional review board in accordance with the United States Department of Health and Human Services regulations at 45CFR46 | IR# 3467-EXT |
| WLHS | The ethical committee in Sweden | Dnr 210-93 |

**File A**: Statistical analysis plan for evaluation of the association between alcohol intake and pancreatic cancer risk within the Pooling Project on Alcohol and Cancer

1. ***Study sample***

The Pooling Project on Alcohol and Cancer (PPAC) is an international consortium of prospective studies conducted within the Pooling Project of Prospective Studies of Diet and Cancer (DCPP). Cohort studies will be included following a set of inclusion criteria to maximize the quality and the comparability across studies:

1. prospective design
2. at least one publication on diet and cancer
3. long-term comprehensive dietary assessment method sufficient to calculate intakes of most nutrients including total energy
4. validation study of the dietary assessment method used in the study or a closely related instrument
5. alcohol intake assessed in grams of ethanol per day
6. that sex-specific sub-cohorts had over 10% alcohol drinkers (alcohol intake ≥ 0.1g/day) at baseline
7. a minimum of 50 incident pancreatic cancer cases were documented during follow-up.

Exclusion criteria will be applied to participants with:

- a prevalent cancer diagnosis at baseline other than non-melanoma skin cancer
- log_e_-transformed energy intake measurements beyond three standard deviations of the study- and sex-specific log_e_-transformed mean energy intake
- missing data on alcohol intake, or alcohol intakes exceeding 200 g/day.

1. ***Exposure assessment***

The primary exposure of interest will be self-reported alcohol intake in grams of ethanol per day. Daily amount of alcohol will be calculated from baseline study-specific questionnaires based on the frequency of consumption, the number of drinks and the alcohol content of the alcoholic beverages consumed at baseline calculated as the sum of beverage-specific intakes, namely beer, wine and spirits/liquor.

Data on alcohol intake and the baseline risk factors will be harmonized across the 30 studies, including smoking habits, weight, height, race, ethnicity, education, physical activity and prevalent diabetes status.

1. ***Outcome assessment***

The outcome will be defined as first primary incident pancreatic cancer cases using the International Classification of Diseases (ICD) code 157 (9^th^ edition) or C25 (10^th^ edition).

Endocrine tumors (ICD-9 code 157.4 and ICD-10 code C25.4) and lymphoproliferative tumours (ICD-Oncology 3rd edition codes: 9251, 9560, 9590, 9591, 9680, 9691, 9695, 9950) will be excluded.

Histologically confirmed cases will be identified excluding ICD-O codes other than 9990 and 9999.

1. ***Statistical analysis***

1. ***Primary analyses***

The association between alcohol intake and pancreatic cancer risk will be evaluated using multivariable Cox proportional hazards models on aggregated individual-level data from each study into a unique dataset. Hazard ratios (HR) and 95% confidence intervals (CI) will be estimated.

The primary time scale will be follow-up time in years, from age at baseline until the age at cancer diagnosis, death, or administrative end of follow-up, whichever occurred first.

The baseline hazard will be stratified by age at recruitment, year of baseline questionnaire, study, country, and sex (in sex-combined models).

Alcohol intake will be modelled:

- in categories as: <0.1, 0.1–<5, 5–<15, 15–<30, 30–<60 and ≥60 g/day, using the group 0.1–<5 g/day as the reference category, to minimize the effect of former heavy drinkers. As women drink less than men, the two highest categories of alcohol intake will be collapsed into a ≥30 g/day group
- in continuous, for a 10 g/day increase, with an indicator to model drinking status (0/1 = <0.1 g/day / >=0.1 g/day)

Covariates to be included in the model will be chosen based on the most recent literature and categorized similarly across studies. We plan to adjust models for:

- detailed smoking habits, including smoking status (never, former, current), smoking duration (in years, coding never smokers as 0), smoking intensity (in number of cigarettes/days, coding 0 for never smokers), time since smoking cessation in past smokers (in years, coding 0 for never and current smokers)
- diabetes status (yes, no)
- body mass index (continuous, kg/m^2^)
- height (continuous, centimetres)
- education level (<high school, high school,> high school)
- self-identified race and ethnicity (African American, Asian, Caucasian, Hispanic, Other)
- physical activity (low, medium, high)
- total energy intake (continuous, kcal/day)

Missing values for covariates will be modelled with missing indicator variables.

Tests for statistical significance of pancreatic cancer HRs related to alcohol intake in categories will be performed with p-values comparing the Wald test statistics to a χ^2^ distribution with degrees of freedom equal to the number of alcohol categories minus one, not including the category of non-drinkers (<0.1 g/day). P-values for trend across categories of alcohol intake will be obtained in models including alcohol intake as a continuous variable.

The associations between alcohol intake from different alcoholic beverages, including beer, wine and spirits/liquors, and pancreatic cancer risk will be assessed in separate models, using the following categories: <0.1 g/day, 0.1–<3 (reference), 3–<10, 10–<20, 20–<40 and ≥40 g/day. These models will be further adjusted for the sum of the alcohol intake from alcoholic beverages other than the one under evaluation.

1. ***Sensitivity analyses***
2. ***Linearity***

We will assess potential departures from linearity in the association between alcohol intake and pancreatic cancer risk using (multivariable) restricted cubic spline models with internal knots placed at the center of each alcohol intake category and using 2.5 g/day as the reference. Participants with alcohol intake greater than 100 g/day will be excluded. Models will include the same list of covariates as for alcohol intake modelled in continuous. Nonlinearity will be evaluated by comparing the difference in log-likelihood of models with linear term and fractional polynomials to a χ^2^ distribution with two degrees of freedoms.

1. ***Heterogeneity***

We will evaluate heterogeneity in the alcohol intake and pancreatic cancer risk association by:

- Study
- Sex
- Smoking status (never, former, current smokers)
- Geographic region (Europe-Australia, North America, Asia)
- BMI (18.5-<25,25-<30≥30 kg/m²)
- Diabetes status (yes, no)
- Education level (≤high school, >high school)
- Multi-vitamin use (yes, no, in North American cohorts)
- Follow-up time (<2, 2-<5, 5-<10, ≥10 years)

Models will include alcohol intake modelled in continuous and the candidate effect modifier as categorical variable, and adding an interaction term between alcohol intake and the effect modifier. P-values for heterogeneity will be obtained by comparing the log-likelihood of models with and without the interaction terms to a χ^2^ distribution with degrees of freedom equal to the number of categories of the effect modifier minus one. Missing values on the candidate effect modifier will result in participant exclusion in models evaluating that modifier. Models will be adjusted as previously described for alcohol intake modelled in continuous.

1. ***Proportional hazard assumption***

Proportional hazards assumption will be evaluated introducing a continuous time dependent variable modelled as the interaction between the log-transformed follow-up time and alcohol intake.

1. ***Residual confounding from smoking***

To evaluate the effect of different adjustments of smoking variables on pancreatic cancer HRs related to alcohol, different models were compared:

- no smoking covariates
- adjustment for smoking status
- further adjustment for smoking duration, smoking intensity, and time since smoking cessation
- other covariates

1. ***Reverse causation***

To assess potential reverse causation, the association between alcohol use and pancreatic cancer risk will be re-evaluated after excluding the first 2 years of follow-up.

1. ***Confirmed pancreatic cancer cases***

The association will be re-examined after restricting the case definition to histologically confirmed pancreatic cancer cases.

1. ***Past drinking***

Among cohorts with information about past drinking, we plan to evaluate the association after separating out former drinkers from never drinkers in the baseline non-drinkers category.

1. ***Overall***

All statistical tests will be two-sided with nominal level of statistical significance set to 5%. Analyses will be performed using SAS version 9.4 (SAS Institute, Cary, NC, USA).

Statistical analysis plan for evaluation of the association between alcohol intake and pancreatic cancer risk within the Pooling Project on Alcohol and Cancer
