# Supplementary material for: Alcohol intake and pancreatic cancer risk: An analysis from 30 prospective studies across Asia, Australia, Europe, and North America
Source: PLoS Med. 2025 May 20;22(5):e1004590. doi: 10.1371/journal.pmed.1004590 (PMC12091891; doi:10.1371/journal.pmed.1004590)
Supplement: Attachment — Submitted filename: PMEDICINE-D-24-02276R1_Answer-to-reviewers-and-editor_final.docx [file pmed.1004590.s005.docx]

**PMEDICINE-D-24-02276R1**

**Answer to Editor and reviewers**

**ACADEMIC EDITOR**

This paper examines the association between alcohol consumption and the risk of pancreatic cancer, a topic of significant global importance. The methodology, including both the study design and analysis, is exceptional, and the results are both interesting and impactful. While previous systematic reviews and meta-analyses on this topic have been published, the strength of the current study lies in its pooling of individual-level data from 31 cohort studies. This approach allows for more sophisticated analyses and provides sufficient power to examine the effects of alcohol consumption at different dose levels, with appropriate control for confounders and the ability to explore effect modification. The inclusion of international cohorts enhances the generalizability of the findings on a global scale. The key findings are of high clinical relevance and could have implications for public policy.

A few comments for the authors:

**Comment 1:**

The analysis explores heterogeneity across the cohort studies by using interaction terms between alcohol intake (modeled continuously) and each study. The results indicate that there was no heterogeneity between the cohorts. However, while heterogeneity was not observed, does this eliminate the need to address clustering by cohort? Alternative approaches to account for clustering should be considered, such as hierarchical models or generalized estimating equations.

**Authors’ answer:**

In line with the Editor’s suggestion, we compared results from analyses testing for heterogeneity, introducing an interaction term between alcohol intake and the variable ‘study’, to a hierarchical multi-level model, that included the variable ‘study’ as a random intercept. Results of models with interaction terms and with random effects were similar with HR estimates for a 10 g/day increase in alcohol intake of 1.03 (95% CI: 1.02, 1.04).

**Comment 2:**

Additionally, the methodology for addressing heterogeneity, particularly heterogeneity by study, is not clearly explained in the statistical analysis section. Although the figure legend describes the approach well, I recommend including more detail in the methods section regarding how heterogeneity was handled.

**Authors’ answer:**

In line with these the recommendations the description of the methodology used to assess heterogeneity by study was updated in Material and Methods, lines 357-370:

“We evaluated heterogeneity in the alcohol intake and pancreatic cancer risk association by study, sex, smoking status (never, former, current smokers), geographic region (Europe-Australia, North America, Asia), BMI (18.5-<25, 25-<30, ≥30 kg/m²), prevalent diabetes status (yes, no), education level (≤high school, >high school), multi-vitamin use (yes, no, in North American cohorts), and follow-up time (<2, 2-<5, 5-<10, ≥10 years). Models included alcohol intake (in continuous), a categorical variable for the candidate effect modifier, and an interaction term between alcohol intake and the effect modifier. P-values for heterogeneity were obtained by comparing the log-likelihood of models with and without the interaction terms to a χ^2^ distribution with degrees of freedom equal to the number of categories of the effect modifier minus one. Participants with missing values of the candidate effect modifier were excluded in models evaluating that modifier and models were adjusted as previously described for alcohol intake modelled in continuous.”

**Comment 3:**

Lastly, the authors mention that data harmonization was performed across the different cohorts, but they do not explain how this was achieved, given that the cohorts likely collected data differently. Further elaboration on the harmonization process would be helpful.

**Authors’ answer:**

Details on data harmonization have been clarified in the new version of the manuscript. Accordingly, we have added the following description:

- for the Study Sample in the Material and Methods, lines 255-259: “Individual-level data from participating cohorts to the DCPP were centralized and harmonized at the Harvard T. Chan School of Public Health (11,12,14,15,19–32,32–43). Details on harmonization are described in sections on exposure assessment, outcome assessment, and statistical analysis”.
- Harmonization of alcohol intake was described in Material and Methods, lines 286-290: “Daily amount of alcohol in grams of ethanol was calculated from the baseline study-specific questionnaires based on the frequency of consumption, the number of drinks and the alcohol content of the alcoholic beverages consumed. All studies had a measure of total alcohol intake at enrolment calculated as the sum of the beverage-specific intakes, namely beer, wine and spirits/liquor.”
- Harmonization of covariates was described in the statistical analysis section in Material and Methods, page 10, lines 324-343.
- Harmonization of outcome assessment was described in Material and Methods, page 9, lines 299-307.

**REVIEWER #1**

**Comment 1:**

This study investigates the association between alcohol drinking and pancreatic cancer risk through a pooled-analyses of 30 prospective studies, allowing the assessment of the possible modifying effect of various factors. The study is very well conducted and presented. I would only modify the main message in the abstract and conclusion, indicating that the study supports an association for high alcohol consumption.

**Authors’ answer:**

We thank the Reviewer for the positive comments. Findings from this evaluation corroborate existing evidence of a positive modest linear association between alcohol intake and pancreatic cancer. When alcohol intake was modeled in continuous for a 10 g/day increase, the pancreatic cancer HR was equal to 1.03 (95% CI: 1.02 to 1.04) and the dose-response analysis showed no evidence for deviation from linearity. These results indicate that, consistently with other sites of cancer, there is no safe dose for alcohol intake. For these reasons, we would be reluctant to emphasize an association between alcohol intake and pancreatic cancer risk restricted to “high alcohol intake”, and would rather support a more cautious interpretation of our results. Nevertheless, we agree with the suggestion to emphasize that a carcinogenic role of alcohol was particularly evident for alcohol intakes of at least 15 and 30 g/day, in women and men respectively. The abstract, author summary, and the conclusions were amended as described below:

- Abstract, page 3, lines 119-122: “Findings from this large-scale pooled analysis support a modest positive association between alcohol intake and pancreatic cancer risk, irrespective of sex and smoking status. Associations were particularly evident for baseline alcohol intake of at least 15g/day in women and 30g/day in men.”
- Authors summary, page 4, lines 149-151: “These findings support a modest positive association of alcohol intake with pancreatic cancer risk, irrespective of sex and smoking status. Associations were apparent for alcohol intake of at least 15 g/day in women and 30 g/day in men”
- Conclusion, page 25, lines 641-646: “Findings from this large consortium of prospective studies support a modest positive association between alcohol intake and pancreatic cancer risk, irrespective of smoking status and sex. Associations were particularly evident for baseline alcohol intake of at least 15 g/day in women and 30 g/day in men.”

**Comment 2:**

Please correct "95%CI" in "95% CI" throughout all the Manuscript.

**Authors’ answer:**

We addressed the Reviewer’s request and applied the change throughout the manuscript.

**Abstract**

**Comment 3:**

The sentence "Alcohol intake was positively associated with pancreatic cancer risk, with HR5-to-<15g/day, ….." can be modified into "Alcohol intake above 30 g/day was positively associated with pancreatic cancer risk, with HR30-to-<60g/day and HR≥60g/day equal to 01.12 (95%CI:1.03,1.21) and 1.32 (95%CI: 1.18,1.47), respectively, compared to intake of 0.1-<5g/day.".

**Authors’ answer:**

The text was amended in line with the Reviewer’s suggestion.

**Comment 4:**

The sentence "specifically in women (HR: 1.03; 95%CI:1.01,1.06; pvalue<0.01) and never smokers (HR: 1.03; 95%CI:1.01,1.06; pvalue<0.01) with no evidence of heterogeneity by sex (pheterogeneity=0.27) or smoking status (pheterogeneity=0.62)" can be modified as follows "No evidence of heterogeneity was observed by sex (p heterogeneity=0.27) or smoking status (p heterogeneity=0.62)".

**Authors’ answer:**

The text in the Abstract, lines 107-111 was amended in line with the Reviewer’s suggestion, and reads as: “A 10 g/day increment of alcohol intake was associated with a 3% increased pancreatic cancer risk overall (HR: 1.03; **95% CI**:1.02,1.04; p_value_<0.01) and among never smokers (HR: 1.03; 95%CI:1.01,1.06; p_value_<0.01), with no evidence of heterogeneity by sex (p_heterogeneity_=0.27) or smoking status (p_heterogeneity_=0.62)”.

**Comment 5:**

The conclusion should be modified as follows "Findings from this large-scale pooled analysis support a modest positive association between elevated alcohol intake (at least 15g/day in women and 30g/day in men) and pancreatic cancer risk, irrespective of sex and smoking status".

**Authors’ answer:**

In line with the Reviewer’s suggestion, the Abstract, lines 119-122, was updated as:

“Findings from this large-scale pooled analysis support a modest positive association between alcohol intake and pancreatic cancer risk, irrespective of sex and smoking status. Associations were particularly evident for baseline alcohol intake of at least 15g/day in women and 30g/day in men.”

**Introduction**

**Comment 6:**

Authors may also quote this pooled analysis of case-control studies "Lucenteforte E et al. Alcohol consumption and pancreatic cancer: a pooled analysis in the International Pancreatic Cancer Case-Control Consortium (PanC4). Ann Oncol. 2012 Feb;23(2):374-82. doi: 10.1093/annonc/mdr120"

**Authors’ answer:**

We thank the Reviewer for the suggestion, the reference was added to the Introduction, lines 221-223.

**Discussion**

**Comment 7:**

Please revise the first sentence of the Discussion and the Conclusion following the previous suggestion.

**Authors’ answer:**

In line with the Reviewer’s suggestion the Conclusions, lines 641-647, reads as follow:

“Findings from this large consortium of prospective studies support a modest positive association between alcohol intake and pancreatic cancer risk, irrespective of smoking status and sex. Associations were particularly evident for baseline alcohol intake of at least 15 g/day in women and 30 g/day in men. These results will inform future experts’ evaluations on the epidemiological evidence of the carcinogenicity of alcohol intake.”

**Tables**

**Comment 8:**

In the Tables, please delete the “%” sign, since this is already indicated in the first column.

**Authors’ answer:**

Table 2 and Supplementary Table 1 were updated accordingly.

**REVIEWER #2**

This is a well-conducted study on the association between alcohol intake and pancreatic cancer risk based on an analysis from 30 prospective studies. The study design, datasets, statistical methods and analyses, and presentation (tables and figures) and interpretation of the results are mostly adequate. However, there are still a few issues needing attention.

**Comment 1:**

1. Justification of inclusion of 30 studies. As we know, the gold standard of pooling evidence is a systematic review and meta-analysis through systematic search of literatures. But this study is not. As the studies included didn't go through systematic search, how its results compared to a meta-analysis? pros and cons? Are there any more recent meta-analysis on this topic?

**Authors’ answer:**

An advantage of pooling projects of primary participant level data is that they can include cohort studies that have not published on the association being examined. On the basis of the inclusion criteria described in the Material and Methods, lines 240-257, cohorts participating in the DCPP were invited to participate in this study. An additional four cohorts were identified through the NCI Cohort Consortium. The text in Material and Methods, lines 253-257 was clarified as:

“Individual-level data from participating cohorts to the DCPP were centralized and harmonized at the Harvard T.H. Chan School of Public Health (11,12,14,15,19–32,32–43). Details on harmonization are described in sections on exposure assessment, outcome assessment, and statistical analysis”.

Unlike meta-analyses that combine summary statistics from published studies, pooled analyses include individual-level data from participating cohorts, irrespective of whether their results were published. Advantages of this approach include:

(1) More flexibility to conduct subgroup analyses, e.g., among never smokers. In meta-analyses, sub-group hazard ratio estimates may not be available, often due to limited power. If sub-group estimates are provided in published studies, it is likely because they are statistically significant, thus resulting in a selected list of included estimates, i.e., publication bias. In this study, pooling individual-level data enabled careful examination of effect modification by smoking status, and several other candidate effect modifiers, to be carried out.
(2) More comparable definition of the exposure, the confounding factors, and the outcome (here pancreatic cancer), as well as of the statistical models, thus reducing potential sources of heterogeneity across studies that are common in meta-analyses.
(3) More inclusive criteria for participating cohorts. Even small cohorts can contribute to consortia.

As a result, our pooled analyses yielded more accurate (and likely precise) estimates compared to meta-analyses of the published literature.

We identified one more recent meta-analysis (PMID: 37905315). However, the study does not list the studies included in the evaluation, nor the number of cohorts included for the analysis. It was not possible to identify to what extent this evaluation overlapped with previous meta-analyses or pooled evaluations.

**Comment 2:**

2. As shown in the limitations in page 23, it says "exposure assessments at baseline generally relates to participants' alcohol intake during mid-adulthood, and do not account for potential within-person change in alcohol intake from adolescence to later in life, nor for changes that may occur after baseline". This is crucially important. We know people's drinking pattern changes from adolescence to mid-life and then later life, but so far this study only captured a static mid-life one. As the included studies varies in starting times and also follow-up times, there are substantial uncertainties in un-captured drinking patterns. Besides, from GBD studies we know people are drinking less and less over the past 3 decades. However, all these trends in drinking were not captured in the study. Therefore, the robustness and reliability of findings are subject to scrutiny.

**Authors’ answer**

As pointed by the Reviewer, alcohol intake was collected at baseline from cancer-free participants (median age at recruitment of 57 years), but information about drinking patterns during the lifetime was not consistently available. We acknowledged this limitation of this study and provided recommendations for future studies in the Discussion, lines 625-631:

“Additionally, alcohol intake evaluated in this study expressed participants’ average intake in grams of ethanol per day over the year preceding baseline, and did not account for alcohol intakes earlier in life, for example during early-adulthood (73). However, in a previous study from the EPIC cohort, baseline and lifetime alcohol intake showed similar positive associations with pancreatic cancer risk (14). In addition, the present study did not evaluate the impact of specific drinking patterns, for example characterised by large amounts over short durations (binge drinking), due to lack of specific information.”

**Comment 3:**

3. Competing risk. As the endpoint of the analysis is pancreatic cancer incidence, death poses a competing risk in the analysis, which has not been addressed in the study.

**Authors’ answer**

The Reviewer is correct that overall mortality exerts a competition to pancreatic cancer in our data. However, under the assumption of non-informative censoring, hazard ratio estimates of pancreatic cancer in relation to alcohol intake are unbiased, unlike estimates of absolute risks.

**REVIEWER #3**

This study addresses an important and timely research question - associations between alcohol consumption and pancreatic cancer where evidence was considered as limited or inconclusive by international expert panels hence updated evidence is needed. This study, with larger sample size, availability of more diverse cohorts, and greater power for subgroup analyses than previous studies, provides updated evidence to help clarify the observational association of alcohol drinking with pancreatic cancer, especially by sex and smoking status. I have several comments below for potential further improvements of the paper.

**Comment 1:**

*	Lines 109-110, "specifically in women…": The dose-response association seems much clearer in men than women (Figure 1), and HR per 10g/day was significant in both sexes. Is there any reason to particularly report women findings in the abstract?

**Authors’ answer**

In line with the Reviewer’s recommendation, we updated the manuscript accordingly in the Abstract, lines 107-111:

“A 10 g/day increment of alcohol intake was associated with a 3% increased pancreatic cancer risk overall (HR: 1.03; 95% CI:1.02,1.04; p_value_<0.01) and among never smokers (HR: 1.03; 95%CI:1.01,1.06; p_value_<0.01), with no evidence of heterogeneity by sex (p_heterogeneity_=0.27,) or smoking status (p_heterogeneity_=0.62)”

**Comment 2:**

*	Lines 298-300, 305-306: An alternative way to quantify the effect of continuous alcohol intake would be to analyse alcohol intake (as continuous variable of g/day) among drinkers only. Have the authors considered this method, over performing the analyses in all participants while adjusting for binary alcohol status as described in the paper?

**Authors’ answer**

Analysis of alcohol drinkers only provided virtually identical results to the models that we presented which included alcohol as continuous and an indicator variable for drinking status.

**Comment 3:**

*	Lines 314-326, following on above comment: are these done among drinkers only, or in all participants but adjusting for binary alcohol status variable?

**Authors’ answer**

All analyses of alcohol intake in continuous also included a binary variable for drinking status. We updated our methods as follows to make it clear for every step of our analyses.

- Material and Methods, lines 353-354:

“Models included the same list of covariates as detailed earlier for alcohol intake modelled in continuous.”

**Comment 4:**

*	Lines 454-456: though p for heterogeneity is significant for wine by education level, the HRs themselves are non-significant. I'd suggest to be cautious and avoid over-interpretation of this heterogeneity results.

**Authors’ answer**

We agreed with the Reviewer and updated the text in the Results, lines 502-504:

“Pancreatic cancer HR estimates for alcohol intake from wine differed by geographic region (Europe/Australia: HR_10g/day_=1.00, 95% CI: 0.96, 1.04; North-America: HR_10g/day_=1.04, 95% CI: 1.00, 1.07; Asia: HR_10g/day_=0.85 95% CI: 0.77, 0.94; p_region_<0.01).”

**Comment 5:**

*	Lines 518-520, Supplementary table 1. The median alcohol intake among drinkers is much higher in Asia than Europe-Australia/North America. The extent of gap is quite surprising. Are the any potential explanations or insights for this gap that could perhaps be discussed in the discussion and support the data?

**Authors’ answer**

The Reviewer pointed out the distinct drinking pattern in Asia compared to other regions of the worlds. The larger prevalence of individuals carrying genetic mutations that control the enzymes that convert ethanol into acetaldehyde among Asians reduce their tolerance to alcohol drinking and lead to lower percentages of alcohol drinkers among participants from Asian cohorts. However, we have no plausible explanation as why the average alcohol intake among Asian consumers was larger than in other areas. In line with the Reviewer’s suggestion, references of studies reporting similar drinking patterns among Asian populations were reported in the Discussion, lines 568-578:

“A larger proportion of participants in the Asian cohorts were non-drinkers at baseline (62% overall, 82% in women, 55% in men) compared to other regions (15% in Europe/Australia and 33% in North America), while the median alcohol intake among male drinkers from Asian cohorts was higher than among drinkers from other regions (Supplementary Table 1). These figures are consistent with previous observations from other Asian cohorts (64,65).”

**Comment 6:**

*	Null associations in Asian studies: while lightly touched upon (e.g. line 507 that Asian cohorts only represented 8% of the study population), the point that Asian studies are underrepresented and uncertainty of the null associations (e.g. potentially due to lack of power?) could perhaps be discussed a little more, or discussed as suggestion for future research/limitation.

**Authors’ answer**

We followed the Reviewer’s recommendations and further extended the discussion regarding the null association observed among Asian cohorts and extended our recommendations for future research:

- Discussion, lines 568-572:

“In our study, genetic data was not available. Asian populations were under-represented, with 5 cohorts out of 30 and a total of 904 incident pancreatic cancer cases included. These figures, together with the large proportion of non-drinkers, particularly among Asian women, might explain the null association observed in Asian cohorts.”

- Discussion, lines 639-642

“Finally, although data from 30 cohorts were pooled in this study, further collaborative efforts are needed to provide more comprehensive evaluations of the alcohol-PC association for specific tumor subtypes, and in geographic regions that were not included or underrepresented in this study.”

**Comment 7:**

*	It would also be good to have a short discussion to pinpoint suggested direction of future studies based on the study findings (also see above comment).

**Authors’ answer**

We addressed the Reviewer’s comment by adding guidance for future investigations in the Discussion, lines 637-642:

“Future studies leveraging longitudinal assessments of alcohol intake from early to mid-adulthood (73) may provide insights on into the impact of alcohol drinking at different ages on pancreatic cancer risk. Finally, although data from 30 cohorts were pooled in this study, further collaborative efforts are needed to develop more comprehensive evaluations of the alcohol-PC association for specific tumor subtypes, and in geographic regions that were not included or underrepresented in this evaluation.”

**Comment 8:**

*	In the Abstract, the conclusion (line 122-123) mentioned that the heterogeneity by regions and beverage types might be due to different drinking habits (and hence further investigation needed), but this idea seemed not presented/discussed in the main text (particularly discussion). It would perhaps be good to introduce or, if appropriate, elaborate this a little considering also other potential explanations (e.g. power/availability of relevant studies for inclusion, differential drinking habits, other confounding factors) in the main text.

**Authors’ answer**

In line with the Reviewer’s recommendation the text was amended:

- Discussion, lines 619-621:

“The study also had limitations. Although pooling individual-level data enabled adjustment for a comprehensive list of confounders, we cannot rule out potential bias from unmeasured confounders.”

- Discussion, lines 625-639:

“Additionally, alcohol intake evaluated in this study expressed participants’ average intake in grams of ethanol per day over the year preceding baseline, and did not account for alcohol intakes earlier in life, for example during early-adulthood (73). However, in a previous study based on the EPIC cohort, baseline and lifetime alcohol intake showed similar positive associations with pancreatic cancer risk (14). In addition, the present study did not evaluate the impact of specific drinking patterns, for example characterised by large amounts over short durations (binge drinking), due to lack of specific information. Future studies leveraging longitudinal assessments of alcohol intake from early to mid-adulthood (73) may provide insights on into the impact of alcohol drinking at different ages on pancreatic cancer risk.”

**General editorial requests**

Note: not all will apply to your paper, but please check each item carefully

**Request 1**

*	We ask every co-author listed on the manuscript to fill in a contributing author statement, making sure to declare all competing interests. If any of the co-authors have not filled in the statement, we will remind them to do so when the paper is revised. If all statements are not completed in a timely fashion this could hold up the re-review process. If new competing interests are declared later in the revision process, this may also hold up the submission. Should there be a problem getting one of your co-authors to fill in a statement we will be in contact. Please do not add or remove authors without first discussing this with the handling editor. You can see our competing interest’s policy here: http://journals.plos.org/plosmedicine/s/competing-interests.

**Authors’ answer**

We confirm that all coauthors were contacted to report their competing interests as indicated in the “conflict of interest statement” (page 26). Furthermore, an excel file that describes each authors contribution and full list of affiliations was uploaded together with the manuscript files.

**Request 2**

*	Please upload any figures associated with your paper as individual TIF or EPS files with 300dpi resolution at resubmission; please read our figure guidelines for more information on our requirements: <http://journals.plos.org/plosmedicine/s/figures>. While revising your submission, please upload your figure files to the PACE digital diagnostic tool, <https://pacev2.apexcovantage.com/>. PACE helps ensure that figures meet PLOS requirements. To use PACE, you must first register as a user. Then, login and navigate to the UPLOAD tab, where you will find detailed instructions on how to use the tool. If you encounter any issues or have any questions when using PACE, please email us at [PLOSMedicine@plos.org](mailto:PLOSMedicine@plos.org).

**Authors’ answer**

Figures 1 to 4 in TIFF format were uploaded with the manuscript files.

**Request 3**

*	Please ensure that the paper adheres to the PLOS Data Availability Policy (see <http://journals.plos.org/plosmedicine/s/data-availability>) which requires that all data underlying the study's findings be provided in a repository or as Supporting Information. For data residing with a third party, authors are required to provide instructions with contact information (web or email address) for obtaining the data. Please note that a study author cannot be the contact person for the data. PLOS journals do not allow statements supported by "data not shown" or "unpublished results." For such statements, authors must provide supporting data or cite public sources that include it.

**Authors’ answer**

The manuscript was updated to report HR estimates instead of results previously described as “data not shown”, page 22, lines 519-521, as: “HRs were also unchanged after excluding the first 2 years of follow-up (HR_10g/day_=1.03, 95% CI: 1.02, 1.04), or after restricting the case definition to histologically confirmed cases (N=9,668 cases; HR_10g/day_=1.03, 95% CI: 1.02, 1.04).”

**Request 4**

*	We expect all researchers with submissions to PLOS in which author-generated code underpins the findings in the manuscript to make all author-generated code available without restrictions upon publication of the work. In cases where code is central to the manuscript, we may require the code to be made available as a condition of publication. Authors are responsible for ensuring that the code is reusable and well documented. Please make any custom code available, either as part of your data deposition or as a supplementary file. Please add a sentence to your data availability statement regarding any code used in the study, e.g. "The code used in the analysis is available from Github [URL] and archived in Zenodo [DOI link]" Please review our guidelines at <https://journals.plos.org/plosmedicine/s/materials-software-and-code-sharing> and ensure that your code is shared in a way that follows best practice and facilitates reproducibility and reuse. Because Github depositions can

be readily changed or deleted, we encourage you to make a permanent DOI'd copy (e.g. in Zenodo) and provide the URL.

**Authors’ answer**

We confirm that the manuscript file now includes a statement that follows editorial request regarding statistical programs availability, page 26, lines 648-649: “Statistical programs can be made available upon request to the corresponding author.”

**Request 5**

*	FINANCIAL DISCLOSURES: The funding statement should include: specific grant numbers, initials of authors who received each award, URLs to sponsors’ websites. Also, please state whether any sponsors or funders (other than the named authors) played any role in study design, data collection and analysis, the decision to publish, or preparation of the manuscript. If they had no role in the research, include this sentence: “The funders had no role in study design, data collection and analysis, decision to publish, or preparation of the manuscript.”

**Authors’ answer**

We confirm that the manuscript file includes funding and acknowledgements statements for the consortium as well as for individual participating cohort, pages 26-31, and follow editorial guidelines specified above.

**Request 6**

*	COMPETING INTEREST: All authors must declare their relevant competing interests per the PLOS policy, which can be seen here: <https://journals.plos.org/plosmedicine/s/competing-interests>

For authors with ties to industry, please indicate whether any of the interests has a financial stake in the results of the current study."

Please add this statement to the manuscript's Competing Interests: "WZ and SCM are Academic Editors on PLOS Medicine's editorial board."

**Authors’ answer**

We confirm that authors’ competing interests were updated according to editorial request, page 26, lines 640-643: “MS is employed at IQVIA, a contract research company, and has no financial stake in the results of the current study. WZ and SCM are Academic Editors on PLOS Medicine's editorial board.”

**Request 7**

*	DATA AVAILABILITY: The Data Availability Statement (DAS) requires revision. For each data source used in your study:

a) If the data are freely or publicly available, note this and state the location of the data: within the paper, in Supporting Information files, or in a public repository (include the DOI or accession number).

b) If the data are owned by a third party but freely available upon request, please note this and state the owner of the data set and contact information for data requests (web or email address). Note that a study author cannot be the contact person for the data.

c) If the data are not freely available, please describe briefly the ethical, legal, or contractual restriction that prevents you from sharing it. Please also include an appropriate contact (web or email address) for inquiries (again, this cannot be a study author).

**Authors’ answer**

We confirm that the manuscript file now include a data availability statement that follows editorial request, page 26, lines 645-648: “The sharing of data will be governed by executed data use agreements between the home institution of the cohort study and the Harvard T.H. Chan School of Public Health, as well as approval from lead investigators for each cohort and/or the cohort study’s leadership.”

**FORMATTING - GENERAL**

**Request 8**

*	Abstract: Please structure your abstract using the PLOS Medicine headings (Background, Methods and Findings, Conclusions). Please combine the Methods and Findings sections into one section.

**Authors’ answer**

We followed PLOS Medicine heading to structure the abstract (Manuscript, page 3)

**Request 9**

*	At this stage, we ask that you include a short, non-technical Author Summary of your research to make findings accessible to a wide audience that includes both scientists and non-scientists. The Author Summary should immediately follow the Abstract in your revised manuscript. This text is subject to editorial change and should be distinct from the scientific abstract. Ideally each sub-heading should contain 2-3 single sentence, concise bullet points containing the most salient points from your study. In the final bullet point of 'What Do These Findings Mean?', please include the main limitations of the study in non-technical language. Please see our author guidelines for more information: <https://journals.plos.org/plosmedicine/s/revising-your-manuscript#loc-author-summary>

**Authors’ answer**

The manuscript file now includes an Authors Summary (page 4).

**Request 10**

*	Please express the main results with 95% CIs as well as p values. When reporting p values please report as p<0.001 and where higher as the exact p value p=0.002, for example. Throughout, suggest reporting statistical information as follows to improve clarity for the reader "22% (95% CI [13%,28%]; p</=)". Please be sure to define all numerical values at first use.

**Authors’ answer**

We confirm to the editor that p-values decimal format was updated through out the manuscript, including figures and tables.

**Request 11**

*	Please include page numbers and line numbers in the manuscript file. Use continuous line numbers (do not restart the numbering on each page).

*	Please cite the reference numbers in square brackets. Citations should precede punctuation.

**Authors’ answer**

We confirm to the editor that pages, lines and references numbers follow editorial instructions

**FIGURES AND TABLES**

*	Please provide titles and legends for all figures and tables (including those in Supporting Information files).

*	Please define all abbreviations used in each figure/table (including those in Supporting Information files).

*	Please consider avoiding the use of red and green in order to make your figure more accessible to those with color blindness.

**Authors’ answer**

We confirm to the editor that figures and tables follow editorial instructions as described above.

**SUPPLEMENTARY MATERIAL**

*	Please note that supplementary material will be posted as supplied by the authors. Therefore, please amend it according to the relevant comments outlined here.

* Please cite your Supporting Information as outlined here: <https://journals.plos.org/plosmedicine/s/supporting-information>

**Authors’ answer**

We confirm to the editor that figures and tables provided as supporting information follow editorial instructions

**REFERENCES**

*	PLOS uses the numbered citation (citation-sequence) method and first six authors, et al.

*	Please ensure that journal name abbreviations match those found in the National Center for Biotechnology Information (NCBI) databases (<http://www.ncbi.nlm.nih.gov/nlmcatalog/journals>), and are appropriately formatted and capitalised.

*	Where website addresses are cited, please include the complete URL and specify the date of access (e.g. [accessed: 12/06/2024]).

*	Please also see <https://journals.plos.org/plosmedicine/s/submission-guidelines#loc-references> for further details on reference formatting.

**Authors’ answer**

Guidelines for reference formatting as provided by the editor were applied to the manuscript.

**STUDY TYPE-SPECIFIC REQUESTS**

*	Abstract: Please include the study design, population and setting, number of participants, years during which the study took place (enrollment and follow up), length of follow up, and main outcome measures.

**Authors’ answer**

We confirm that the abstract includes information requested.

*	Please ensure that the study is reported according to the STROBE (or appropriate STOBE extension) guideline (available from: <https://www.equator-network.org/reporting-guidelines/strobe>) and include the completed STROBE (or STROBE extension) checklist as Supporting Information. Please add the following statement, or similar, to the Methods: "This study is reported as per the Strengthening the Reporting of Observational Studies in Epidemiology (STROBE) guideline (S1 Checklist)." When completing the checklist, please use section and paragraph numbers, rather than page numbers.

**Authors’ answer**

The STROBE checklist was completed and uploaded together with the manuscript files on the PLOS Medicine website.

*	For all observational studies, in the manuscript text, please indicate: (1) the specific hypotheses you intended to test, (2) the analytical methods by which you planned to test them, (3) the analyses you actually performed, and (4) when reported analyses differ from those that were planned, transparent explanations for differences that affect the reliability of the study's results. If a reported analysis was performed based on an interesting but unanticipated pattern in the data, please be clear that the analysis was data driven.

**Authors’ answer**

We confirm that information about hypothesis, planed methods and analyses and results are all described in the manuscript.

*	Please state in the Methods section whether the study had a prospective protocol or analysis plan. If a prospective analysis plan (from your funding proposal, IRB or other ethics committee submission, study protocol, or other planning document written before analyzing the data) was used in designing the study, please include the relevant document(s) with your revised manuscript as a Supporting Information file to be published alongside your study and cite it in the Methods section. A legend for this file should be included at the end of your manuscript. If no such document exists, please make sure that the Methods section transparently describes when analyses were planned, and when/why any data-driven changes to analyses took place. Changes in the analysis, including those made in response to peer review comments, should be identified as such in the Methods section of the paper, with rationale.

**Authors’ answer**

We confirm that the methods section describes transparently the analyses planed and performed.
