# Supplementary material for: Alcohol intake and pancreatic cancer risk: An analysis from 30 prospective studies across Asia, Australia, Europe, and North America
Source: PLoS Med. 2025 May 20;22(5):e1004590. doi: 10.1371/journal.pmed.1004590 (PMC12091891; doi:10.1371/journal.pmed.1004590)
Supplement: Attachment — Submitted filename: PMEDECINE-D-24-02276R2_Answer-to-editor_revision2.docx [file pmed.1004590.s006.docx]

**PMEDECINE-D-24-02276R2 Revision 2**

**Comments from Reviewers**

**Reviewer #2:**

Thanks authors for their effort to improve the manuscript. I am satisfied with the response and revision. No further issues needing attention.

**Reviewer #3:**

The authors have addressed my previous comments satisfactorily. I have no further comments or concerns at this stage

**Requests from Editors**

**GENERAL**

**Request #1**

The terms gender and sex are not interchangeable (as discussed in <https://www.who.int/health-topics/gender#tab=tab_1> ); please use the appropriate term and revise accordingly throughout the manuscript. Please note that you currently use both.

**Answer #1**

The entire manuscript and supplementary material were checked and we confirm that the word “sex” was used throughout the manuscript in its correct context, as reference to individuals’ biological sex, according to the guidelines. The word “gender” was not used.

**FINANCIAL DISCLOSURE**

**Request #2**

Please remove the Acknowledgments section from the Financial Disclosure section of the online submission form. You may include the Acknowledgments at the end of the main manuscript, which will appear as a separate section in the final publication.

**Answer #2**

We removed the acknowledgments from the Financial Disclosure section in both the online form and the manuscript file.

**DATA AVAILABILITY**

**Request #3**

We currently feel that it is not 100% clear how others may request data. Please note that we require you to provide an appropriate contact (web or email address) for requests (this cannot be a study author). Please clarify whether the contact at the Harvard T.H. Chan School of Public Health will be the only point of contact for data requests for any of the study cohorts.

**Answer #3**

The data availability statement was updated as:

“Consortium data can be made available upon request to the EPIC website (https://epic.iarc.who.int/contact-us/). The sharing of data will be governed by executed data use agreements between the home institution of each cohort studies and the Harvard T.H. Chan School of Public Health, as well as approval from lead investigators for each cohort and/or the cohort study’s leadership.”

**TITLE**

**Request #4**

If you agree, we suggest changing the title to "Alcohol intake and pancreatic cancer 1 risk: an analysis from 30 prospective studies across Asia, Australia, Europe, and North America" to reflect the broad scope of your study.

**Answer #4**

We agree with this suggestion of title, just please, remove the “1” between “cancer” and “risk”.

**ETHICS**

**Request #5**

We ask that you provide a list of institutional review boards and corresponding approval numbers. This can be included in the Supplementary Material and referenced in the main text.

**Answer #5**

We updated the supplementary information file with a Table B including names of the institutions. Collection of approval number for each cohort is ongoing We will provide an updated list as soon as possible.

**ABSTRACT**

**Request #6**

1) ll.98/99: PLOS Medicine prefers the use of patient-centered language. Please revise throughout the manuscript.

**Answer #6**

“cancer free participant” was changed to “participants without cancer at baseline”.

**Request #7**

2) l.99: “alcohol drinkers” – we suggest adding ‘(alcohol intake ≥ 0.1g/day)’ here.

**Answer #7**

We have included the suggestion.

**Request #8**

3) l.105ff: Throughout, we suggest reporting statistical information as follows to improve clarity for the reader "1.12 (95% CI [1.03,1.21])".

**Answer #8**

We have included the suggestion in the abstract and throughout the manuscript.

**Request #9**

4) In the last sentence of the Abstract Methods and Findings section, please describe the main limitation(s) of the study's methodology.

**Answer #9**

We moved the following sentence from the Conclusions to the Methods and Findings section: “The differential associations across geographic regions and types of alcoholic beverages might reflect differences in drinking habits and deserve more investigations.”

**Request #10**

5) Please ensure that all numbers presented in the abstract are present and identical to numbers presented in the main manuscript text.

**Answer #10**

We confirm that the numbers presented in the abstract are identical to those presented in the main manuscript.

**Request #11**

6) Please include the important dependent variables that are adjusted for in the analyses.

**Answer #11**

We added the important dependent variables to the abstract as follow:

“In age- and sex-stratified Cox proportional hazards models adjusted for smoking history, diabetes status, body mass index, height, education, race and ethnicity, and physical activity, pancreatic cancer hazard ratios (HR) and 95% confidence intervals (CI) were estimated for categories of alcohol intake and in continuous for a 10 g/day increase. Potential heterogeneity by sex, smoking status, geographic regions, and type of alcoholic beverage was investigated.”

**AUTHOR SUMMARY**

**Request #12**

ll.128-129: Since the Author Summary serves as a non-technical summary of your research in order to make the results accessible to a wide audience, including scientists and non-scientists, we suggest adding a brief note on what 30 g of alcohol per day is equivalent to, e.g. 10 oz or 300 ml of table wine.

**Answer #12**

We updated the authors summary as:

“Previous prospective investigations suggested a harmful role of alcohol in relation to pancreatic cancer development, particularly for alcohol intakes greater than 30g/day, corresponding to daily intake of about 2 standard drinks of either beer, wine or liquors/spirits”.

**INTRODUCTION**

**Request #13**

1) Please change the heading from ‘Background’ to ‘Introduction’.

**Answer #13**

Done.

**Request #14**

2) Please cite the reference numbers in square brackets. Please revise throughout.

**Answer #14**

Done.

**Request #15**

3) l.224: Please define ‘US’ at first use.

**Answer #15**

“US” was defined at first use

**METHODS AND RESULTS**

**Request #16**

1) Table 1: Please define ‘SD’ (l.266). Also, when reporting percentiles, please separate upper and lower bounds with commas instead of hyphens as the latter can be confused with reporting of negative values. Please revise throughout the manuscript.

**Answer #16**

The change was made.

**Request #17**

2) l.323ff: We think it would be easier for the reader if you provided not only the percentage, but also the numerator and denominator. Please revise throughout where necessary.

**Answer #17**

Given the size of the study denominator for each variable it will require to report several million participants. Reporting the fraction instead of the proportion of missing values will decrease the readability of the methods. We prefer not to make this change.

**Request #18**

3) l. 326: Please define ‘BMI’ at first use.

**Answer #16**

The change was made.

**Request #19**

4) Table 2 (and where applicable): Please present numerators and denominators for percentages. When presenting the median, please also provide 10th–90th percentiles.

**Answer #19**

The changes were implemented in Table 2. Given the size of the study and to maximize readability of the table, only numerators were reported

**Request #20**

5) Figure 1/2/3/4: Please define BMI, HR and CI. Please add a reference to the EPIC study.

**Answer #20**

The change was implemented.

**Request #21**

6) Figure 1/2/4 and Table 1: We think that for each category, the included studies should be listed, e.g. in a footnote, as was done for Figure 3, and the relevant references should be provided.

**Answer #21**

Reference for each study was provided in the methods (paragraph 1) and updated in the footnotes of Table 1. However, the data used in this pooled analysis may not reflect data presented in previous publications. We think it could be misleading for readers who may confuse the work with a meta-analysis of previously published results. For this reason, we prefer not to report references on Figures 1/2/3/4.

**Request #22**

7) Figure 2/4: Could you explain why you decided to include the number of studies in Figure 1 and 3 but not in Figure 2 and 4?

**Answer #22**

The results presented in Figure 2 and 4 were estimated in analyses including all cohorts, to the exception of ATBC (a cohort of current smokers at baseline) and NYSC (a cohort where alcohol intake was not collected by type of alcoholic beverages) in Figure 2 and 4, respectively. To clarify, we updated the figures adding a column with the number of studies for each group and described exclusions in the footnotes of each Figure.

**Request #23**

8) Figure 3: We suggest adding the corresponding reference numbers for the studies listed in the figure description.

**Answer #23**

The change was made in Figure 3.

**Request #24**

9) Please include a brief explanation of why Australia was grouped with Europe, as we believe you did not include an explanation for this in the Methods section.

**Answer #24**

We provide explanations for grouping the Australian cohort (MCCS) with European cohorts in the methods l.365-368:

“In analyses by geographical region, the Australian cohort (Melbourne Collaborative Cohort Study, [MCCS]) was combined with European cohorts as participants were mostly Caucasian and reported similar alcohol intake as European cohorts”.

**DISCUSSION**

**Request #25**

1) l.573: Please temper claims of primacy of results by stating, "to our knowledge" or something similar.

**Answer #25**

We updated the manuscript discussion as: “To our knowledge, these results are novel and shed light on inconclusive findings from previous large studies conducted in North America, Europe and Asia”

**Request #26**

2) Please remove any subheadings. The conclusion should be a continuous part of the discussion.

**Answer #26**

The change was made.

**REFERENCES**

**Request #27**

Where website addresses are cited, please use the word ‘accessed’ to specify the date of access (e.g. [accessed: 12/06/2024]).

**Answer #27**

The change was made.

**SUPPLEMENTARY MATERIAL**

**Request #28**

1) Thank you for providing the STROBE checklist. Please replace the page numbers with paragraph numbers per section (e.g. "Methods, paragraph 1"), since the page numbers of the final published paper may be different from the page numbers in the current manuscript.

**Answer #28**

The change was made.

**Request #29**

2) In the published article, supporting information files are accessed only through a hyperlink attached to the captions. For this reason, you must list captions at the end of your manuscript file. You may include a caption within the supporting information file itself, as long as that caption is also provided in the manuscript file. Do not submit a separate caption file.

When SI files are contained with a single file:

Please label the file as ‘S1 Supporting Information’.

Please apply alphabetical labelling to each table and figure contained within the S1 file. For example, ‘Fig A’ to ‘Fig Z’ and ‘Table A’ to ‘Table Z’.

Plain text does not need to be labelled and can just be given a title as necessary. For example, ‘Statistical Analysis Plan’.

Please cite tables/figures as ‘Fig A in S1 Supporting Information’ and/or ‘Table A in S1 Supporting Information’, for example.

Please cite plain text as, ‘Statistical Analysis Plan in S1 Supporting Information’, for example.

When SI files are uploaded as separate files:

Please label tables as ‘S1 Table’ (so on) and figures as ‘S1 Fig’ (and so on).

Any additional documents (protocols/analysis plans etc.) can be labelled as ‘S1 Protocol’, for example. Please cite items as exactly as labelled.

**Answer #29**

The changes were made with Supplementary Information uploaded in a single file. References in the text were also updated.

**General Editorial Requests**

**Request #30**

1) We ask every co-author listed on the manuscript to fill in a contributing author statement. If any of the co-authors have not filled in the statement, we will remind them to do so when the paper is revised. If all statements are not completed in a timely fashion this could hold up the re-review process. Should there be a problem getting one of your co-authors to fill in a statement we will be in contact. YOU MUST NOT ADD OR REMOVE AUTHORS UNLESS YOU HAVE ALERTED THE EDITOR HANDLING THE MANUSCRIPT TO THE CHANGE AND THEY SPECIFICALLY HAVE AGREED TO IT.

**Answer #30**

We confirm that the last version of co-authors list in the manuscript is correct and definitive. Each co-author affiliation(s) and contributions are provided in the additional file “coauthors_list.xls”

**Request #31**

2) Please ensure that the paper adheres to the PLOS Data Availability Policy (see <http://journals.plos.org/plosmedicine/s/data-availability>, which requires that all data underlying the study's findings be provided in a repository or as Supporting Information. For data residing with a third party, authors are required to provide instructions with contact information for obtaining the data. PLOS journals do not allow statements supported by "data not shown" or "unpublished results." For such statements, authors must provide supporting data or cite public sources that include it.

**Answer #31**

We confirm that all results reported are presented according to the PLOS Data Availability Policy, and are reported in the manuscript figures, tables and supplementary information.

**Request #32**

3) Please review your reference list to ensure that it is complete and correct. If you have cited papers that have been retracted, please include the rationale for doing so in the manuscript text, or remove these references and replace them with relevant current references. Any changes to the reference list should be mentioned in the rebuttal letter that accompanies your revised manuscript.

**Answer #32**

We confirm that the references are correct and up-to-date.
