# Supplementary material for: Alcohol intake and pancreatic cancer risk: An analysis from 30 prospective studies across Asia, Australia, Europe, and North America
Source: PLoS Med. 2025 May 20;22(5):e1004590. doi: 10.1371/journal.pmed.1004590 (PMC12091891; doi:10.1371/journal.pmed.1004590)
Supplement: Attachment — Submitted filename: PMEDECINE-D-24-02276R3_Answer-to-editor_revision3.docx [file pmed.1004590.s007.docx]

**PMEDECINE-D-24-02276R3**

**Requests from Editors:**

**Request #1**
1) Ethics statement: Please note that we cannot accept the manuscript until you have provided a complete list of the institutional review boards together with the relevant approval numbers. Again, this can be added in the Supplementary Material and referenced in the main text.

Answer: We understand and are making our best to collect them all. SI Table B provides the most up-to-date information, with pending confirmation for one cohort.

**Request #2**
2) Author Summary: We feel that the main limitation could be more clearly stated in the last bullet point. Please revise.

Answer:

We further clarified the main limitations in the Author Summary as:

“This observational study examined alcohol intake evaluated at a single time point during mid-to-late adulthood and included a limited number of Asian cohorts.  Further research is needed to evaluate the role of lifetime alcohol consumption on pancreatic cancer risk, as well as the impact of specific drinking patterns, including binge drinking.”

**Request #3**
3) Table 2: Please clarify how you calculated the % values for the number of PC cases. For most of the characteristics it seems that the denominator is the total number of male or female participants in the respective group. However, this is not the case for number of PC cases. Please add a short explanation below the table that for categorical variables the denominator for each group of alcohol intake is the number of participants in the corresponding group and explain the exception for number of PC cases. Please also define "PC" below the table.

Answer:

We confirm that the denominator of the percentage of pancreatic cancer cases in each alcohol category is the total number of cases in men and women, separately. We corrected the mistake made in the calculation of the percentage of cases across alcohol intake categories in men and in sex-specific total percentages. We confirm that the rest of the table is correct.

Additionally, the footnotes now describe the meaning for “PC” abbreviation and detail for percentage calculation as:

- “Median and 10th-90th percentile range for continuous variables; numbers and sex-specific percentage for categorical variables.”

**Request #4**
4) Funding: Please include a short statement in the Financial Disclosure Statement that full information on the funding of the original studies (not specifically related to this analysis) can be found in the Supporting Information files. Therefore, please include the additional funding information as a Supporting Information file.

Answer: We respectfully request that the financial disclosure statement could include the full funding information for both the consortium and the individual studies. This is a collaborative project and each study contributed data, provided input during data harmonization, and participated to the interpretation of results.

**Request #5**
5) Please state in the Methods section whether the study had a prospective protocol or analysis plan. If a prospective analysis plan (from your funding proposal, IRB or other ethics committee submission, study protocol, or other planning document written before analyzing the data) was used in designing the study, please include the relevant document(s) with your revised manuscript as a Supporting Information file to be published alongside your study and cite it in the Methods section. A legend for this file should be included at the end of your manuscript. If no such document exists, please make sure that the Methods section transparently mentions that a study protocol is not available and describes when analyses were planned, and when/why any data-driven changes to analyses took place. Changes in the analysis, including those made in response to peer review comments, should be identified as such in the Methods section of the paper, with rationale.

Answer:

We updated the Methods section, line 396-398, as:

“No specific study protocol is available. After data harmonization, a statistical analysis plan was developed jointly between scientists at IARC and at the Harvard T.H. Chan School of Public Health.”

**Technical requests**

**Request #6**

1) Thank you for stating "Participants provided informed consent before they completed questionnaires at baseline."

Please include a statement that formal consent was obtained (must state whether verbal/written) OR the reason consent was not obtained (e.g. anonymity).

NOTE: Please insert an Ethics Statement at the beginning of your Methods section, under a subheading 'Ethics Statement'. It must include:

i) The name(s) of the Institutional Review Board(s) or Ethics Committee(s)

ii) The approval number(s), or a statement that approval was granted by the named board(s)

iii) A statement that formal consent was obtained (must state whether verbal/written) OR the reason consent was not obtained (e.g. anonymity). NOTE: If child participants, the statement must declare that formal consent was obtained from the parent/guardian.]"

Answer: Done.

**Request #7**

2) Please ensure that the funders and grant numbers match between the Financial Disclosure field and the Funding Information tab in your submission form. Note that the funders must be provided in the same order in both places as well.

Currently, the grant number "GR-IARC-2015-05-05-01" is missing from the Funding Information

Answer: Done.

**Request #8**

3) We do not publish any copyright or trademark symbols that usually accompany proprietary names, eg (R), (C), or TM (e.g. next to drug or reagent names). Please remove all instances of trademark/copyright symbols throughout the text, including ©,® on page no 8, ® on page no 9

Answer: Done.
